# Supplementary material for: NCLX prevents cell death during adrenergic activation of the brown adipose tissue
Source: Nat Commun. 2020 Jul 3;11:3347. doi: 10.1038/s41467-020-16572-3 (PMC7334226; doi:10.1038/s41467-020-16572-3)
Supplement: Supplementary file 1 — Supplementary Information [file 41467_2020_16572_MOESM1_ESM.pdf]

Supplementary Information

NCLX prevents cell death during adrenergic activation of the brown adipose tissue

Assali et al. *Nature Communications*. 2020

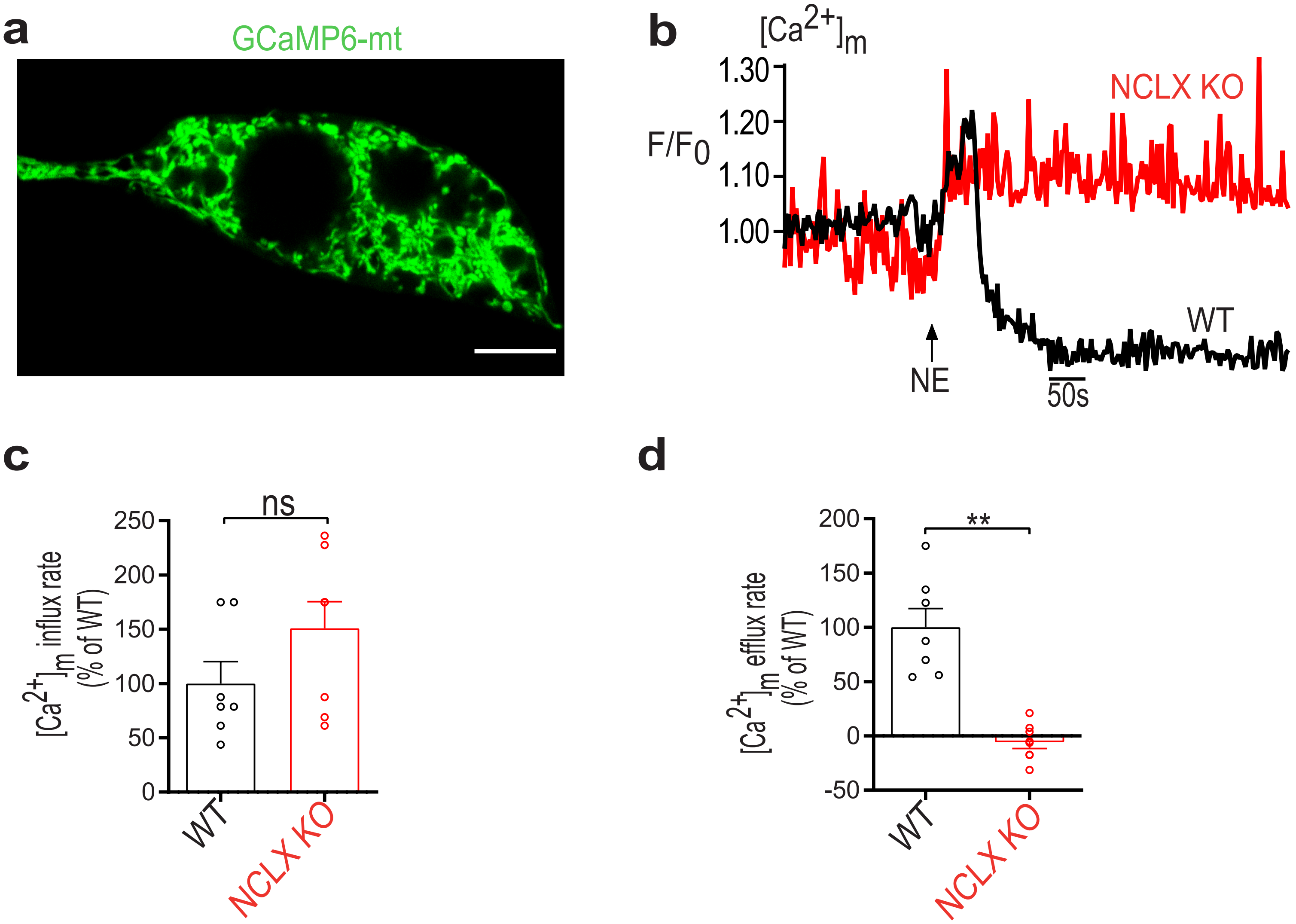

Supplementary Figure 1: Adrenergic stimulation induces mitochondrial Ca^{2+} overload in NCLX KO BA monitored by GCaMP6-mt sensor. (a) Representative super-resolution image of primary WT BA transduced with the mitochondria-targeted Ca^{2+} sensor, GCaMP6-mt. **(b)** Representative mitochondrial Ca^{2+} transients monitored using GCaMP6-mt sensor in NCLX KO and WT BA stimulated with NE. **(c,d)** Quantification of Ca^{2+} influx and efflux rates in NCLX KO and WT BA (n = 7-8 experiments per condition). Student's t-test **(c,d)**. Data are expressed as means ± SEM. ns $p > 0.05$, ** $P<0.01$. Source data are provided as a Source Data file.

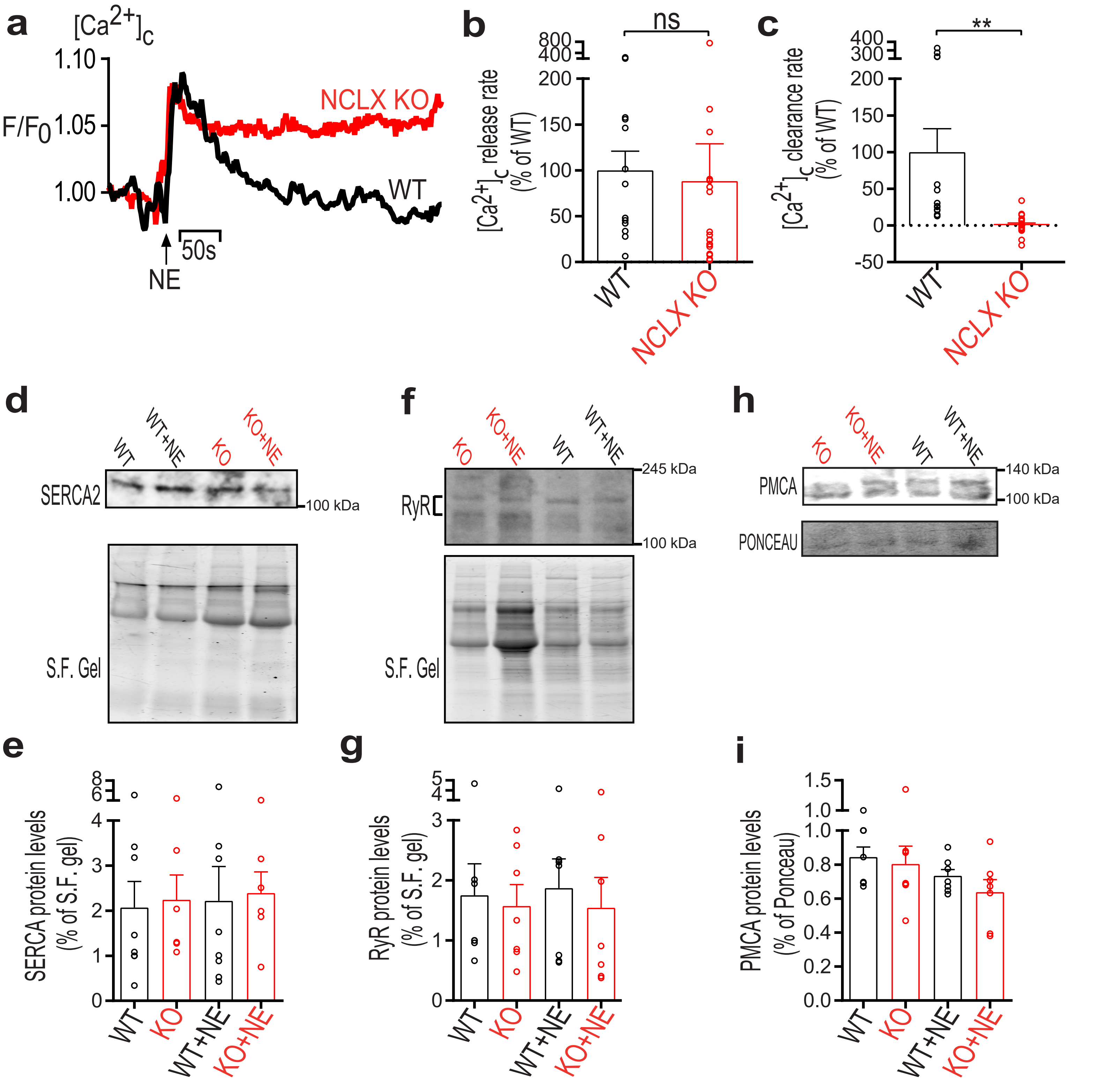

Supplementary Figure 2: Adrenergic stimulation leads to impaired intracellular Ca^{2+} clearance rate in NCLX KO BA. (a) Representative traces of cytosolic Ca^{2+} transients monitored using Fura-2AM fluorescent probe in NCLX KO and control WT BA stimulated with NE. **(b,c)** Quantification of cytosolic Ca^{2+} release and clearance rates in NCLX KO and WT BA (n = 13-18 experiments per condition). **(d)** Representative western blots for SERCA2 in NCLX-null and WT BA. Stain-Free Gel (S.F. Gel) was used as a normalization reference for total protein loading. **(e)** Quantification of SERCA2 levels normalized to S.F. Gel (n = 6-8 per condition). **(f)** Representative western blots for RyR in NCLX-null and WT BA. S.F. Gel was used as a normalization reference for total protein loading. **(g)** Quantification of RyR levels normalized to S.F. Gel (n = 7 per condition). **(h)** Representative western blots for PMCA in NCLX-null and WT BA. Ponceau staining was used as a normalization reference for total protein loading. **(i)** Quantification of PMCA levels normalized to Ponceau (n = 7 per condition).

Student's t-test **(b,c)**; One-way ANOVA with Tukey's post-hoc test **(e,g,i)**. Data are expressed as means ± SEM. ns $p > 0.05$, ** $P<0.01$. Source data are provided as a Source Data file.

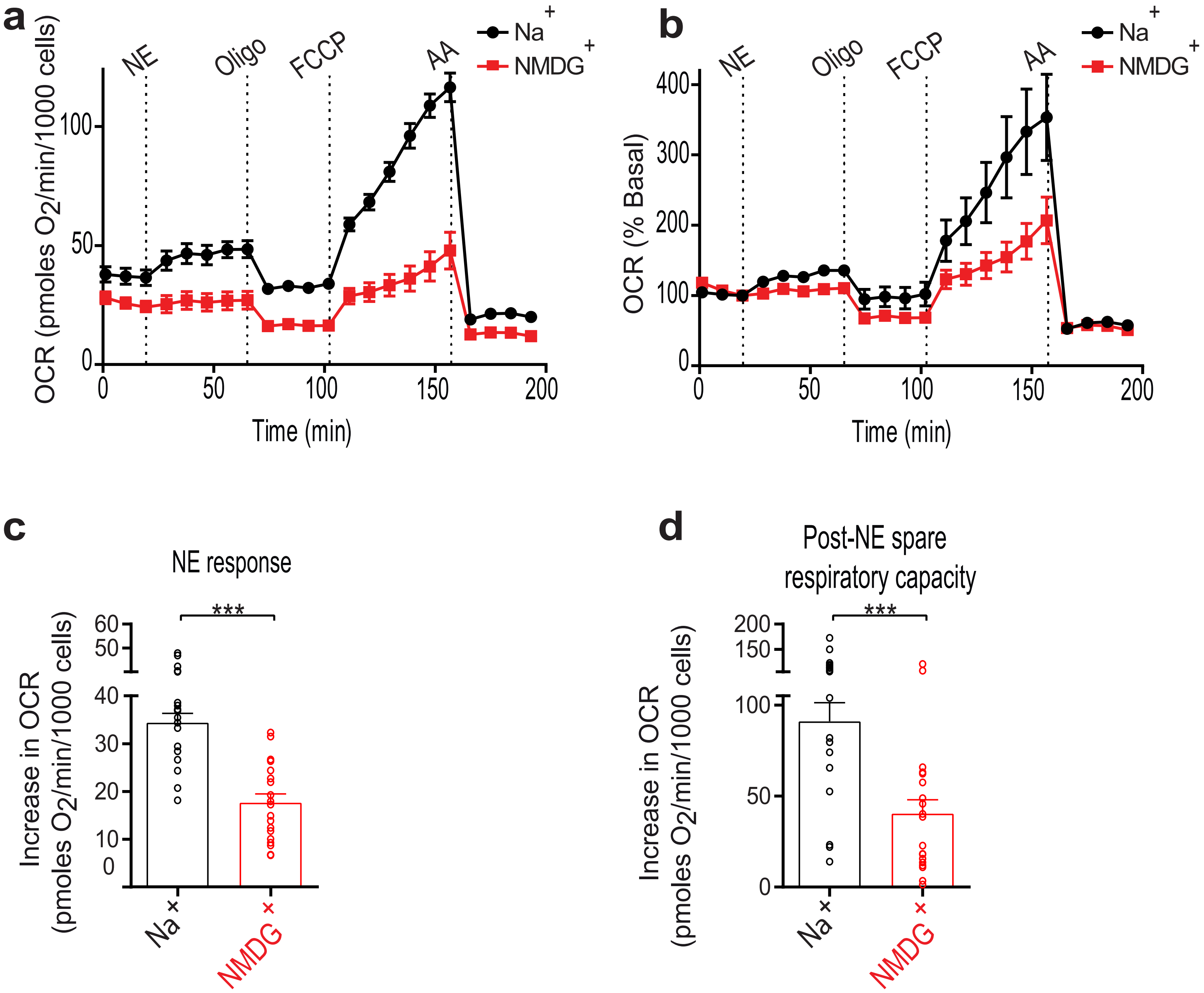

Supplementary Figure 3: Lack of Na^+ impairs NE-stimulated respiration in BA. (a) Representative oxygen consumption rate (OCR) of BA incubated either with or without Na^+ ($NMDG^+$ is used as a cationic replacement) normalized to cell number. **(b)** Representative OCR traces normalized to basal respiration before NE addition. **(c,d)** Quantification of NE response and spare respiratory capacity after NE stimulation of BA incubated with or without Na^+. (N = 3 independent experiments with n = 18-19 total wells per condition). Student's t-test **(c,d)**. Data are expressed as means ± SEM. *** $p < 0.0001$. Source data are provided as a Source Data file.

a

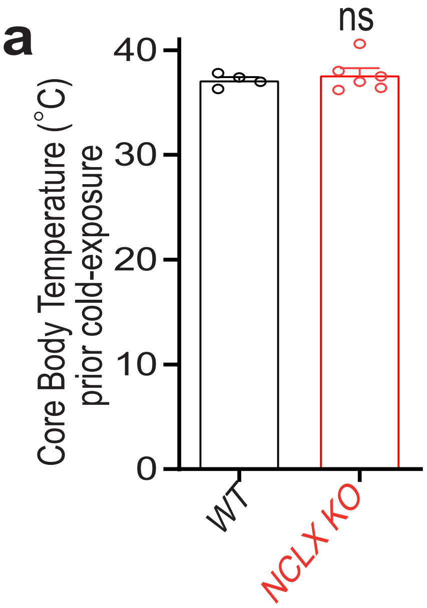

Supplementary Figure 4: Core body temperature of NCLX KO and WT mice under basal conditions. (a) Core body temperature of NCLX KO and WT mice prior to cold-stress at 4°C (n=4-6 mice per group).

Student's t-test **(a)**. Data are expressed as means ± SEM. ns $p > 0.05$. Source data are provided as a Source Data file.

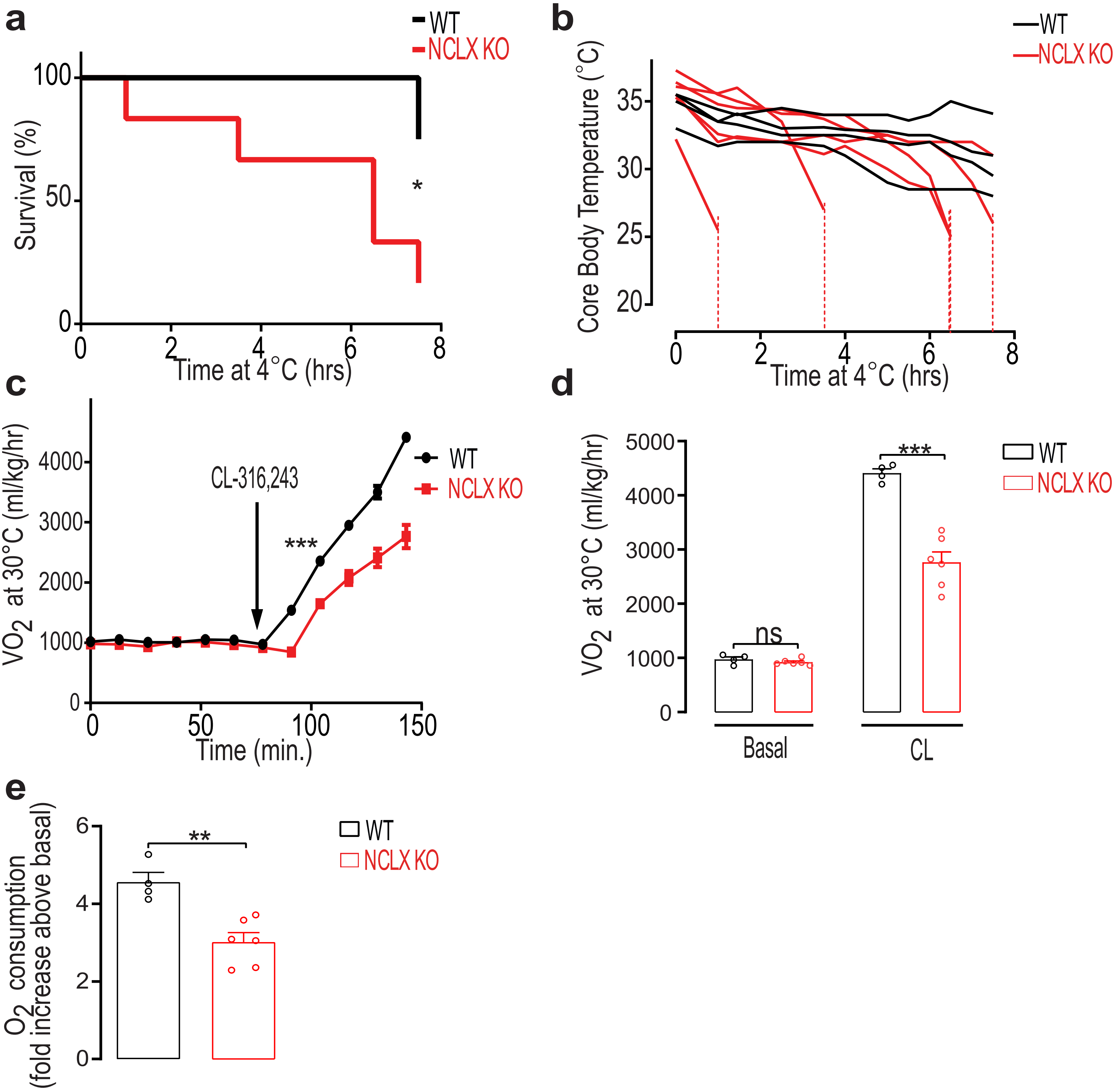

Supplementary Figure 5: Assessment of cold tolerance and non-shiverig thermogenesis in NCLX KO and WT male littermates. (a) Cold tolerance tests at 4°C of NCLX KO and WT male littermates with their raw core body temperature traces and survival curves; Mice reaching 28°C or lower were returned to room temperature for recovery (n = 4-6 mice per group). **(b)** Core body temperature traces of the animals from the experiment shown in (a) during cold exposure (4°C). **(c)** VO2 traces of male littermates of NCLX KO and WT mice at basal and after CL316, 243-injection (1 mg/kg), under anesthesia at 30°C (n = 4-6 mice per group). **(d)** Quantification of VO2 at baseline and under CL-stimulation. **(e)** Fold increase of O2 consumption after the CL-stimulation.

Student's t-test **(d,e)**; Two-way ANOVA **(c)**; Log-Rank statistics **(a)**. Data are expressed as means ± SEM. ns p > 0.05, * P<0.05, ** P<0.01, *** p < 0.0001. Source data are provided as a Source Data file.

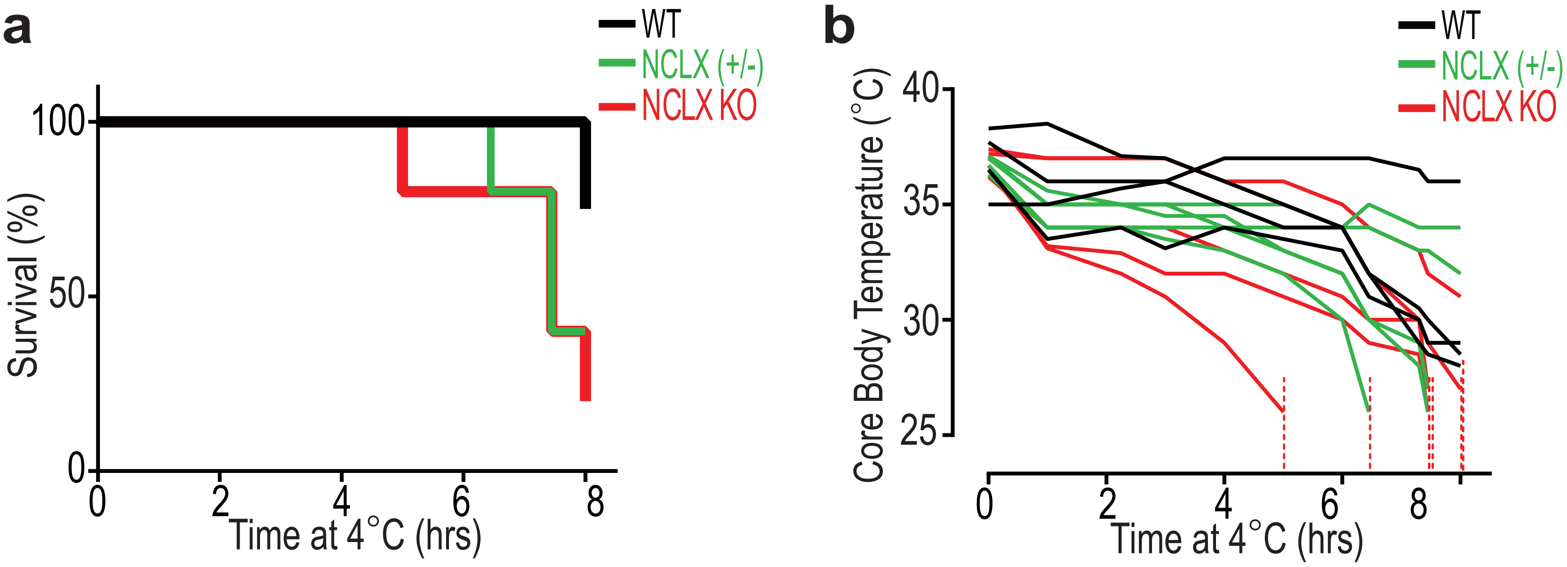

Supplementary Figure 6: Assessment of cold tolerance in NCLX KO, NCLX +/- and WT female littermates.

(a) Survival curves of 8-10 week old females of WT, NCLX +/- and NCLX KO mice cold-stressed at 4°C. Mice reaching 28°C or lower were returned to room temperature for recovery. **(b)** Core body temperature traces of the animals from the same experiment in (a) during cold exposure (4°C).

a

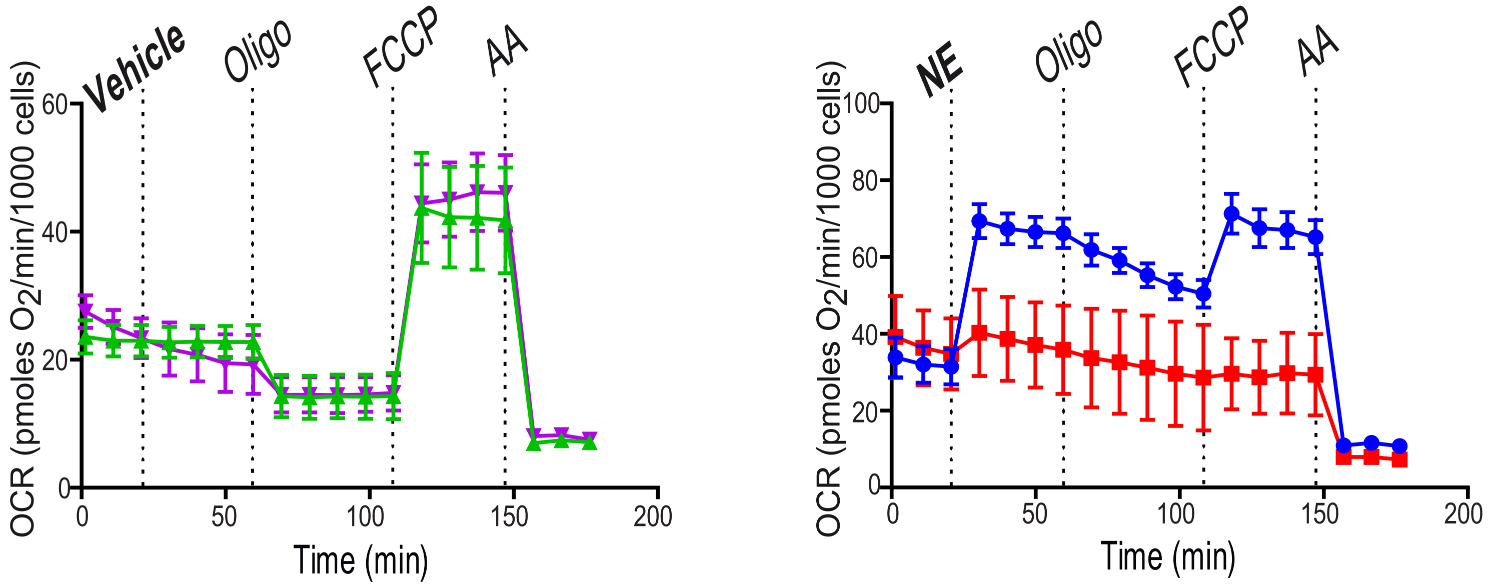

Supplementary Figure 7: Loss of NCLX leads to impaired spare respiratory capacity in BA after adrenergic stimulation. (a) Representative OCR traces of cultured BA from NCLX KO and WT mice. The groups were either stimulated by NE (right graph) or a vehicle (left graph) in the first injection. OLIGOmycin was used to assess mitochondrial uncoupling efficiency. FCCP was then injected to assess maximal respiration and non-mitochondrial OCR was evaluated by Antimycin A injection (AA). Note that only stimulated NCLX KO BA have reduced adrenergic stimulated OCR followed by an impaired spare respiratory capacity that was unaffected in the vehicle non-stimulated NCLX KO BA. Source data are provided as a Source Data file.

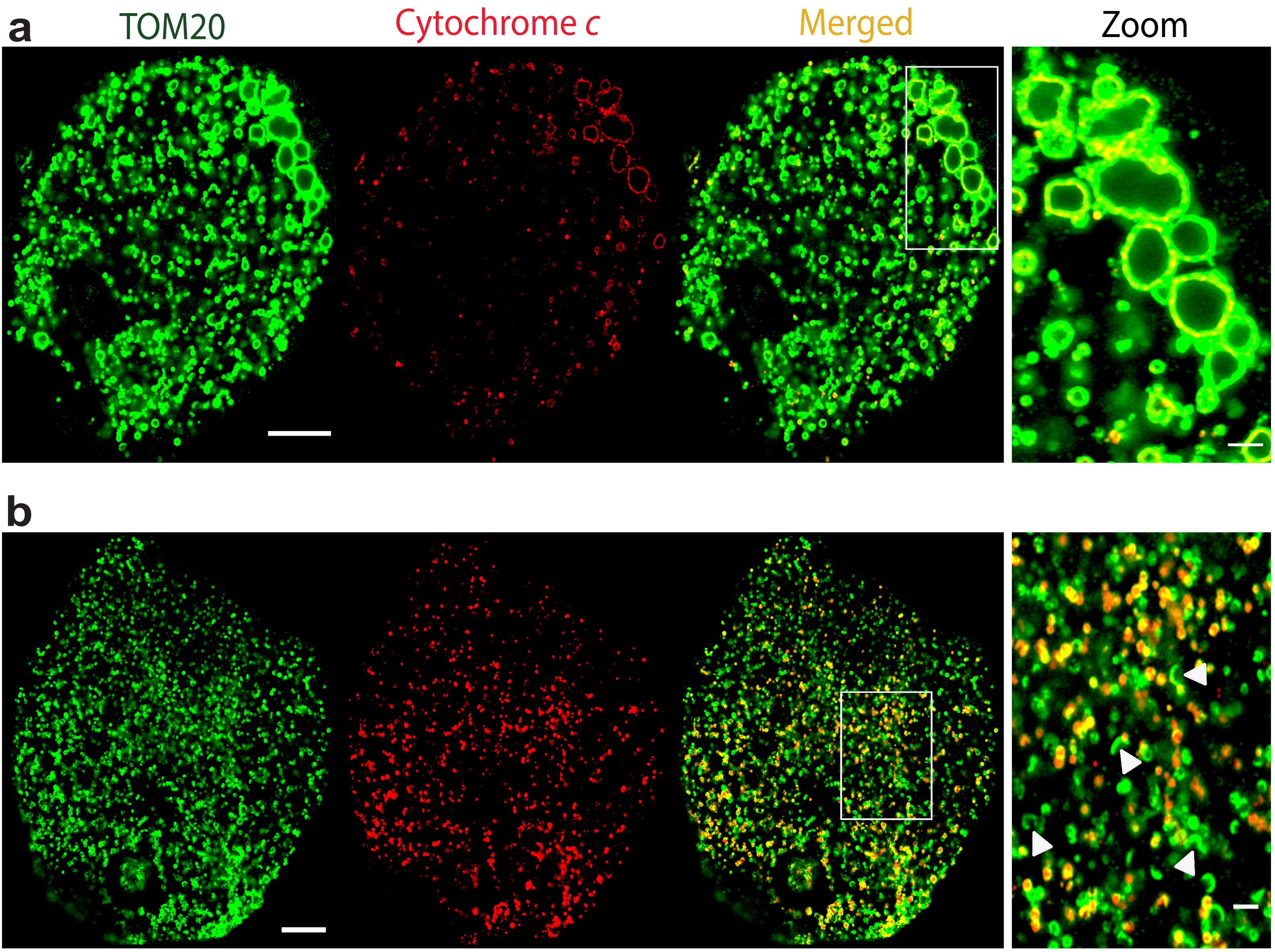

Supplementary Figure 8: Super-resolution imaging for mitochondrial swelling and membrane rupture of stimulated NCLX KO BA. (a,b) Super-resolution confocal images for NE-stimulated NCLX KO BA, co-immunostained for TOM20 for marking mitochondrial network (Green) and for Cytochrome *c* (Red). Note that in NCLX KO BA, in addition to mitochondrial swelling and cytochrome *c* loss, NE induces morphological changes to mitochondria including the rupture of the mitochondrial membranes as indicated by the white arrows.

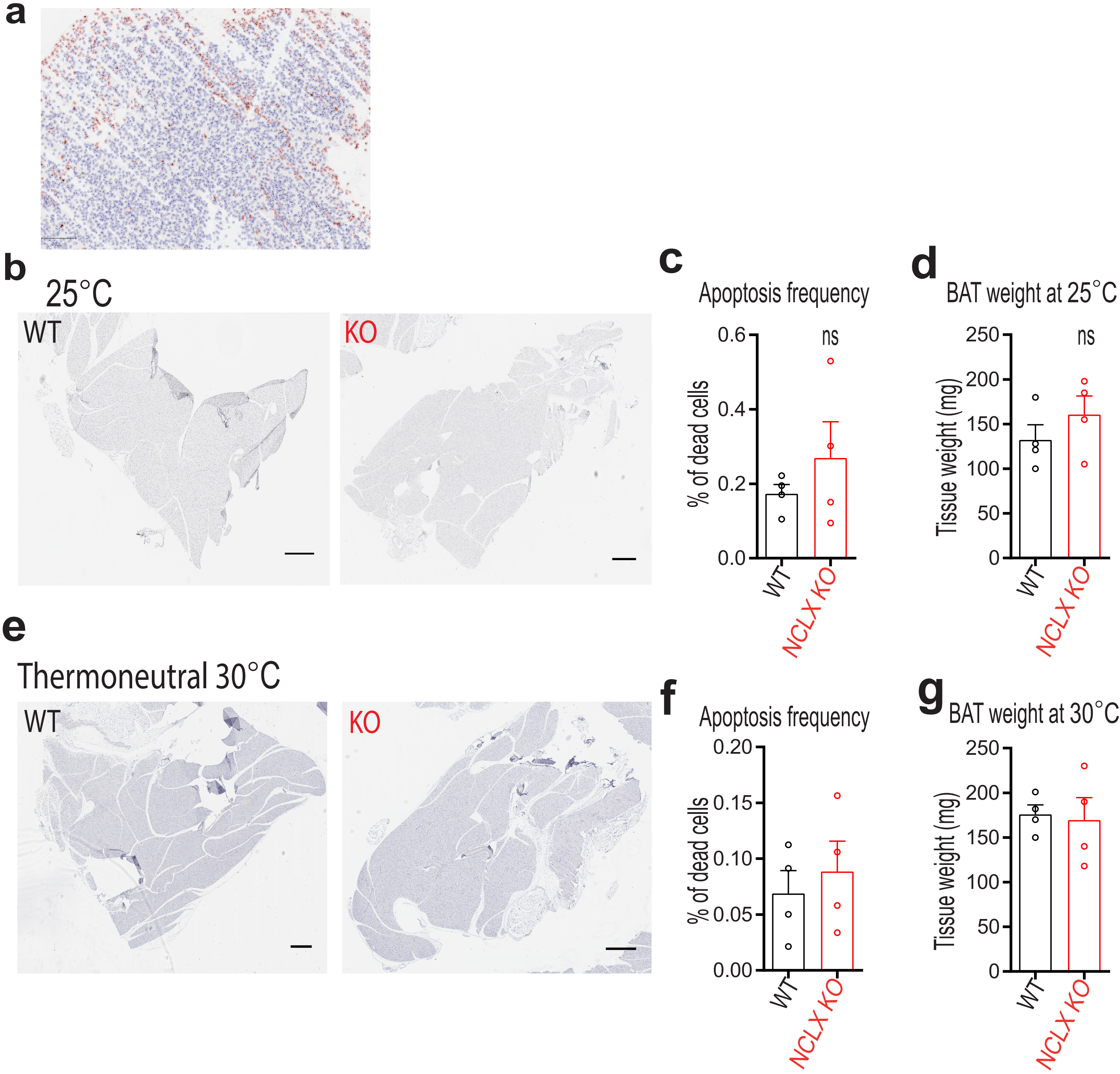

Supplementary Figure 9: BAT phenotyping of NCLX KO and WT mice housed at Room temperature and Thermoneurality. **(a)** Representative of the quantification method of positive dead cells in histology. Red circles indicate positive cells and Blue circles indicate negative cells. **(b)** Cleaved Caspase-3 (CC3) staining of BAT from NCLX KO and WT mice housed at room temperature of 25°C. scale bar, 500 µm. **(c)** Quantification of CC3-positive percentage (n = 4 each group). **(d)** Weight of BAT excised from NCLX KO and WT mice housed at room temperature (n = 4 each group). **(e)** CC3 staining of BAT from NCLX KO and WT mice housed at thermoneutrality (30°C). scale bar, 500 µm. **(f)** Quantification of CC3-positive percentage (n = 4 each group). **(g)** Weight of BAT excised from NCLX KO and WT mice housed at thermoneutrality (n = 4 each group).
Student's t-test **(c,d,f,g)**. Data are expressed as means ± SEM. ns $p > 0.05$. Source data are provided as a Source Data file.

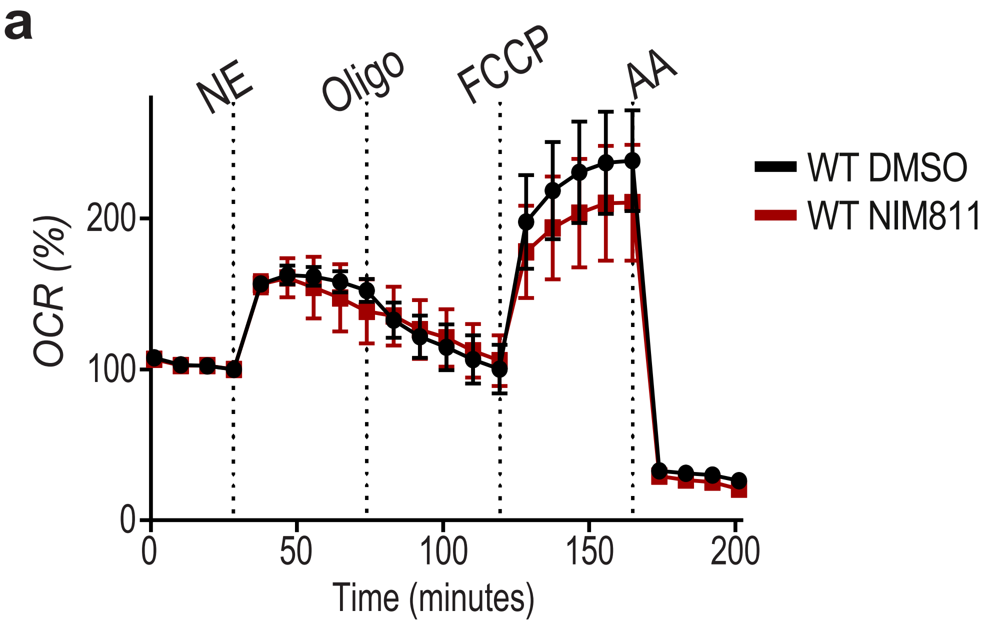

Supplementary Figure 10: Respirometry of WT BA pretreated with NIM811 (a) Representative OCR in response to NE in BA from WT. Cells were pretreated either with DMSO (control) or NIM811 (500nM). Note that NIM811 does not alter respiration in WT BA. Source data are provided as a Source Data file.

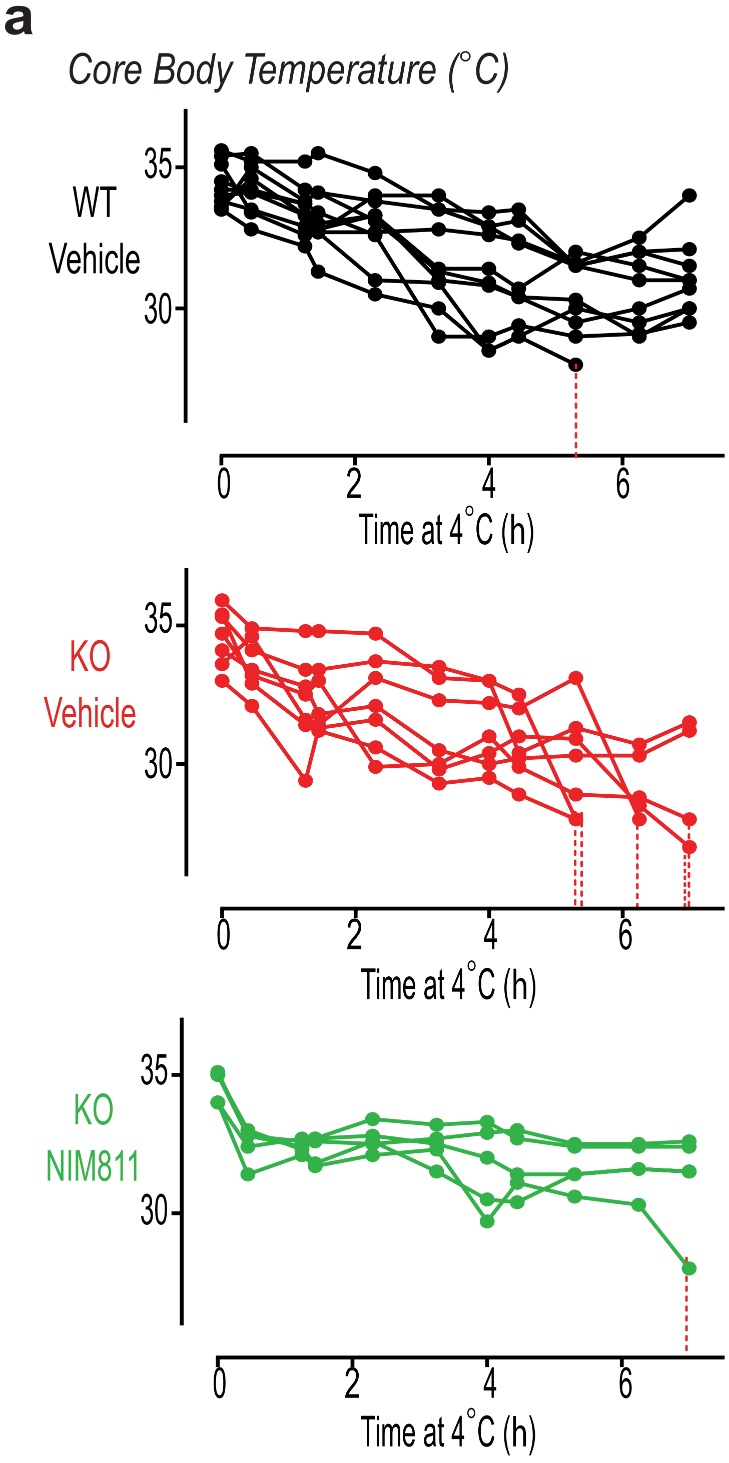

Supplementary Figure 11: Cold tolerance assessment of NCLX KO and WT mice pretreated with or without NIM811. (a) Core body temperature traces during cold exposure (4°C) of the animals from the experiment shown in Figure 7j. Note that the NCLX KO animals display impaired thermogenic capacity as compared to WT, however NCLX KO mice pretreated with NIM811 were protected. The dashed red line indicates animal removal from 4°C.

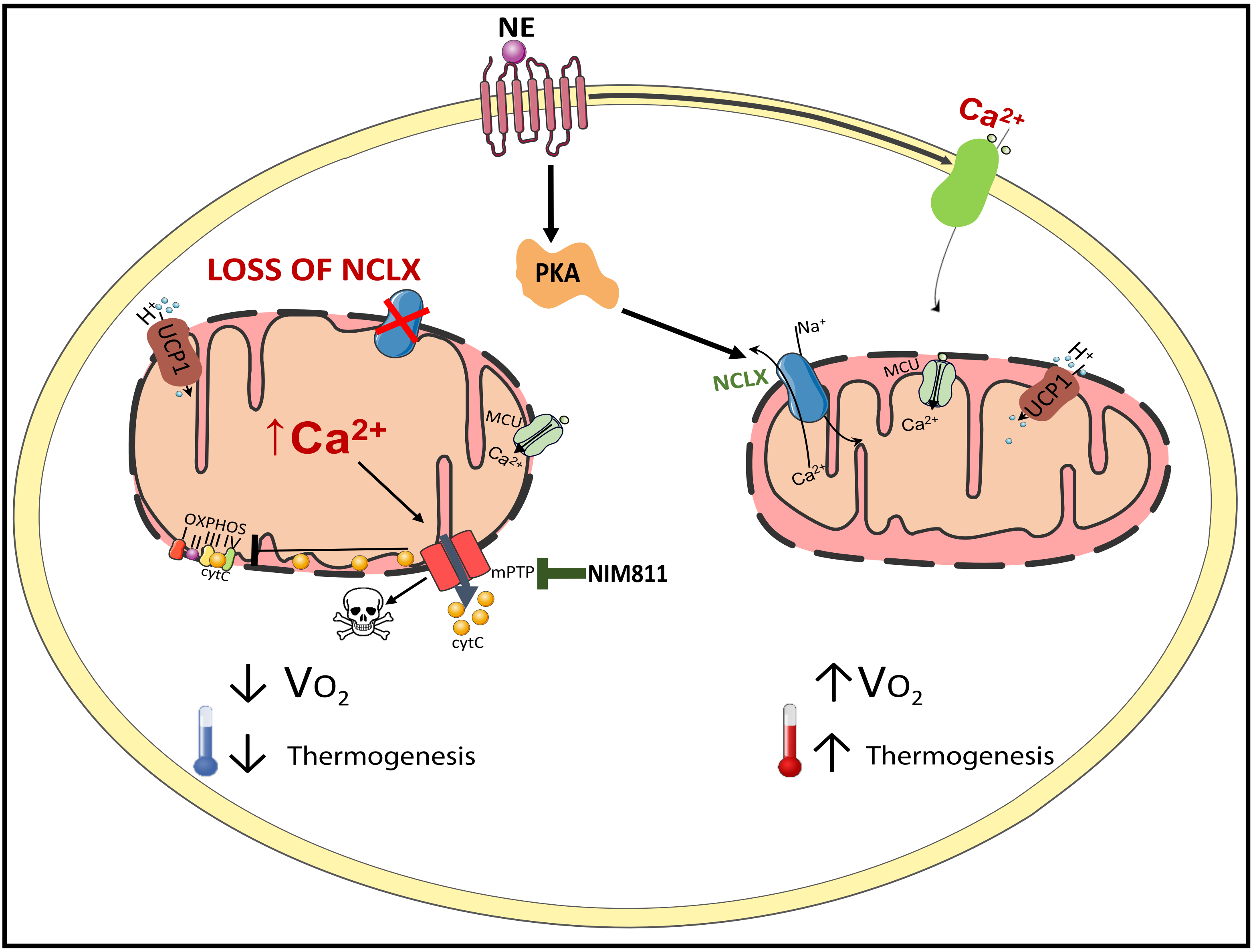

Supplementary Figure 12: Graphical abstract for the role of NCLX under adrenergic activation of BAT. Full description in Figure 8 and the Discussion section.
