# Supplementary material for: NCLX prevents cell death during adrenergic activation of the brown adipose tissue
Source: Nat Commun. 2020 Jul 3;11:3347. doi: 10.1038/s41467-020-16572-3 (PMC7334226; doi:10.1038/s41467-020-16572-3)
Supplement: Supplementary file 3 — Description of Additional Supplementary Files [file 41467_2020_16572_MOESM3_ESM.pdf]

Description of Additional Supplementary Files

File Name: Supplementary Data 1
Description: Source data of high-resolution imaging depicting mitochondrial swelling in stimulated NCLX KO brown adipocytes.

File Name: Supplementary Movie 1
Description: Related to Figure 7. Video of 18F-FDG- µPET/CT Imaging of cold-induced glucose uptake in a representative WT mouse pretreated with vehicle.

File Name: Supplementary Movie 2
Description: Related to Figure 7. Video of 18F-FDG- µPET/CT Imaging of cold-induced glucose uptake in a representative NCLX KO mouse pretreated with vehicle.

File Name: Supplementary Movie 3
Description: Related to Figure 7. Video of 18F-FDG- µPET/CT Imaging of cold-induced glucose uptake in a representative NCLX KO mouse pre-treated with NIM811.
