# Supplementary figures and images for: NCLX prevents cell death during adrenergic activation of the brown adipose tissue
Source: Nat Commun. 2020 Jul 3;11:3347. doi: 10.1038/s41467-020-16572-3 (PMC7334226; doi:10.1038/s41467-020-16572-3)

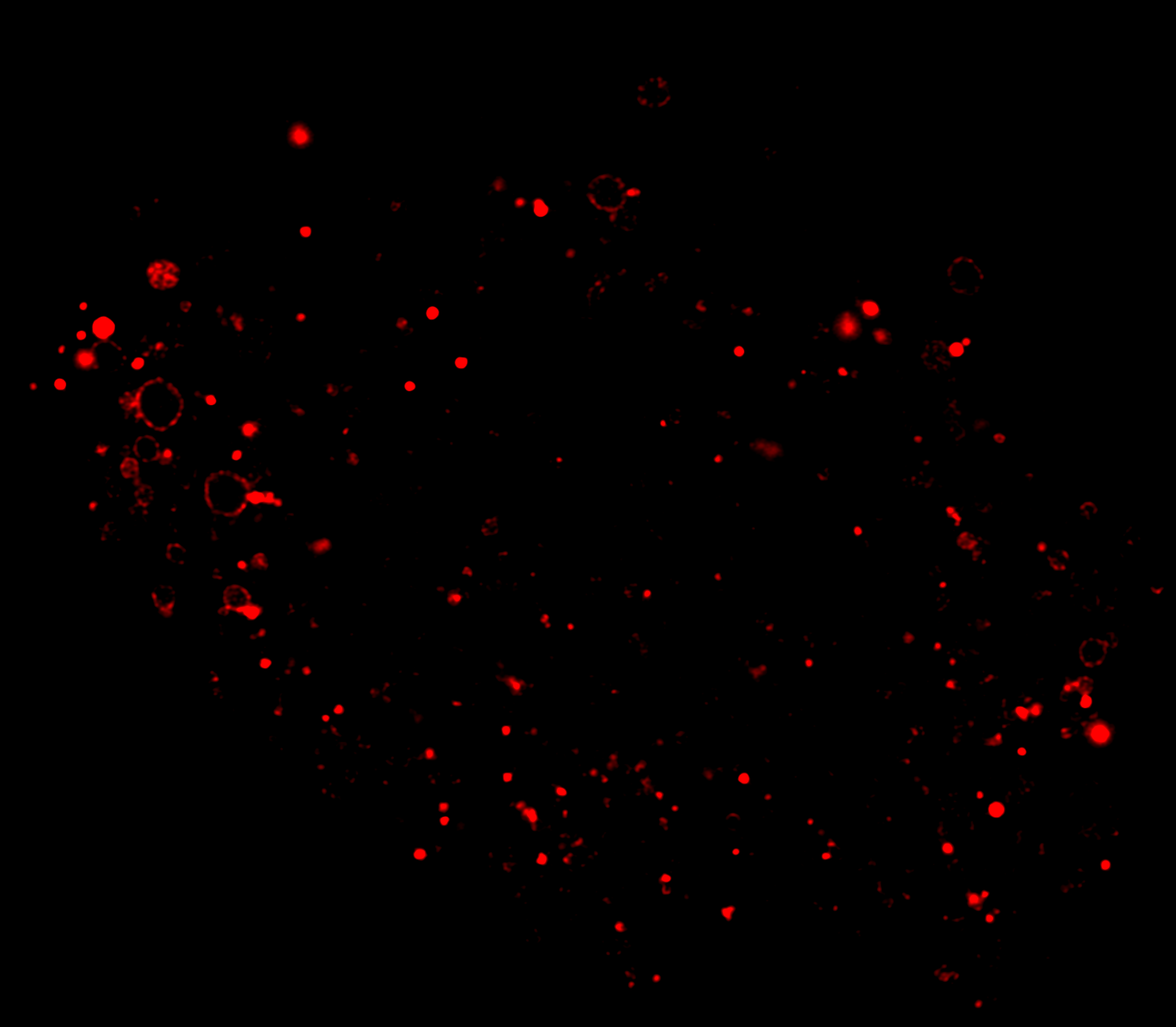

Supplement: Supplementary file 4 — Supplementary Data 1 [file 41467_2020_16572_MOESM4_ESM.zip › Super-Res. Images/Fig. 5a/NCLX KO+NE (Cyt. c).tif]

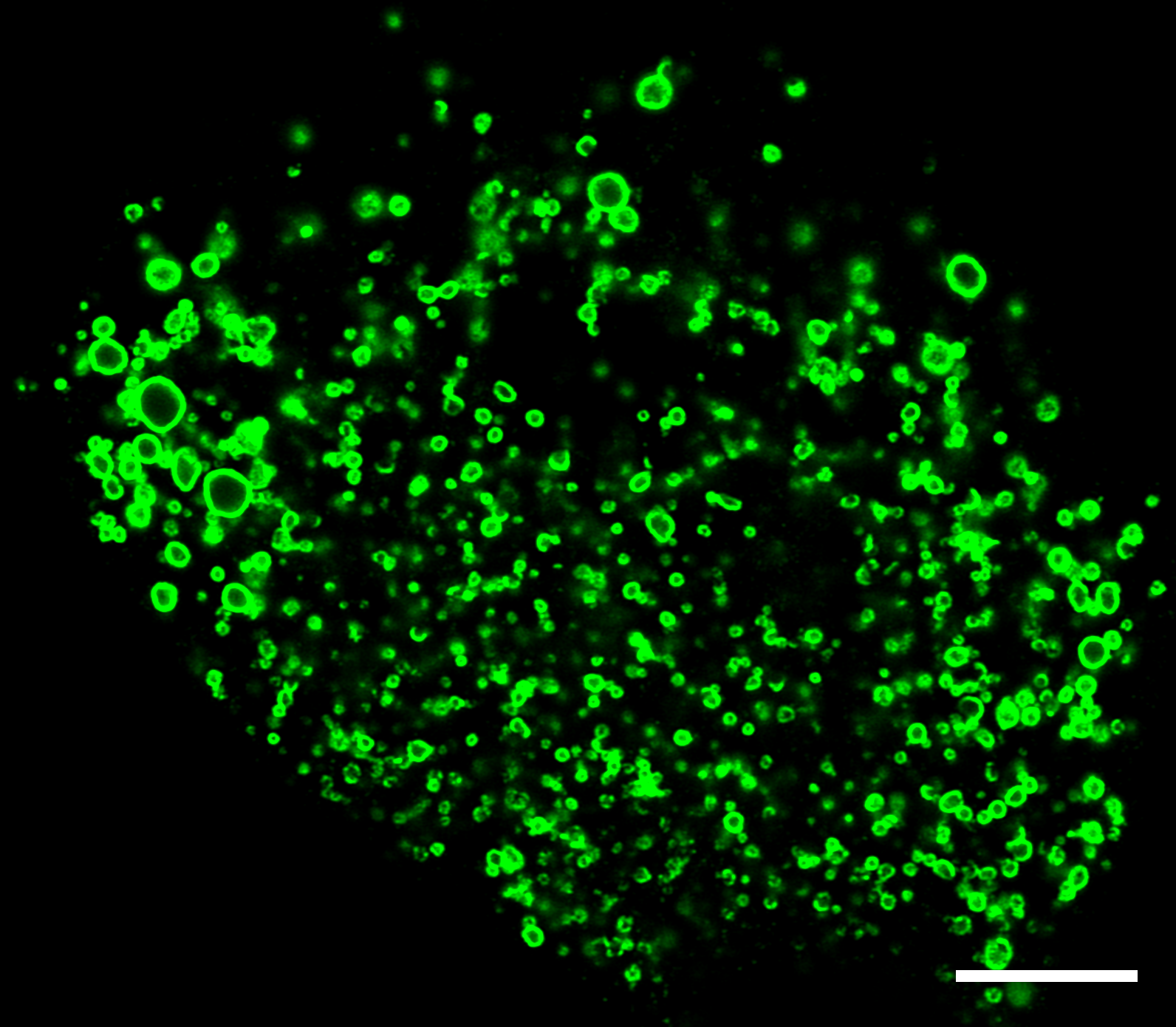

Supplement: Supplementary file 4 — Supplementary Data 1 [file 41467_2020_16572_MOESM4_ESM.zip › Super-Res. Images/Fig. 5a/NCLX KO+NE (TOM20).tif]

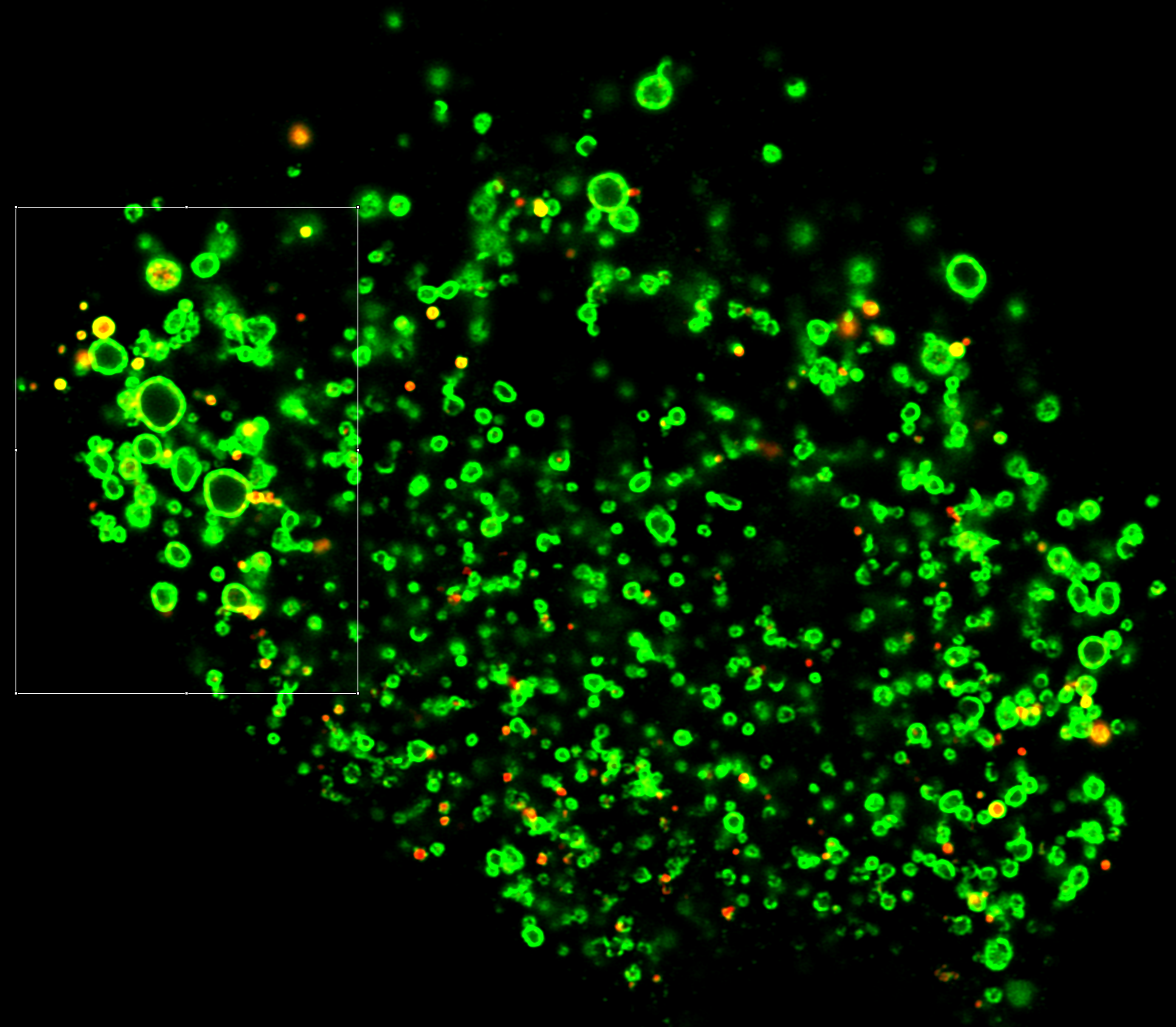

Supplement: Supplementary file 4 — Supplementary Data 1 [file 41467_2020_16572_MOESM4_ESM.zip › Super-Res. Images/Fig. 5a/NCLX KO+NE Composite.tif]

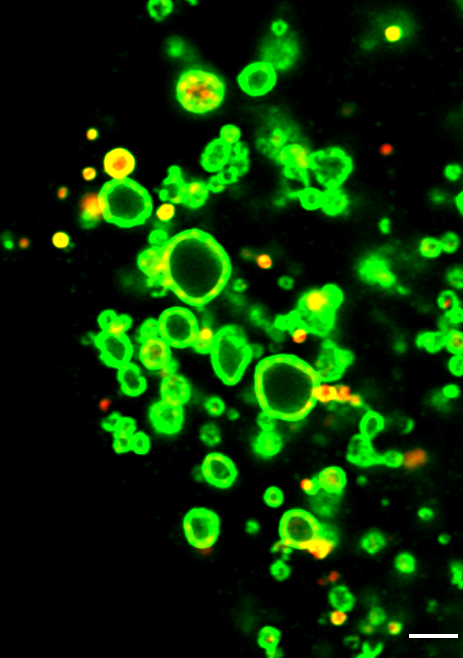

Supplement: Supplementary file 4 — Supplementary Data 1 [file 41467_2020_16572_MOESM4_ESM.zip › Super-Res. Images/Fig. 5a/NCLX KO+NE Zoom.tif]

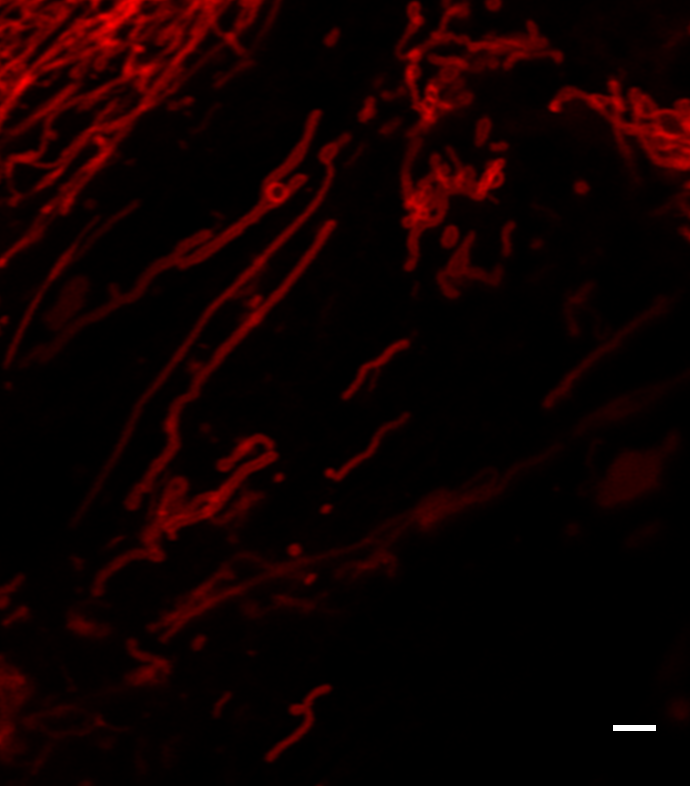

Supplement: Supplementary file 4 — Supplementary Data 1 [file 41467_2020_16572_MOESM4_ESM.zip › Super-Res. Images/Fig. 5d/NCLX KO+NE/NCLX KO t-1 ZOOM.tif]

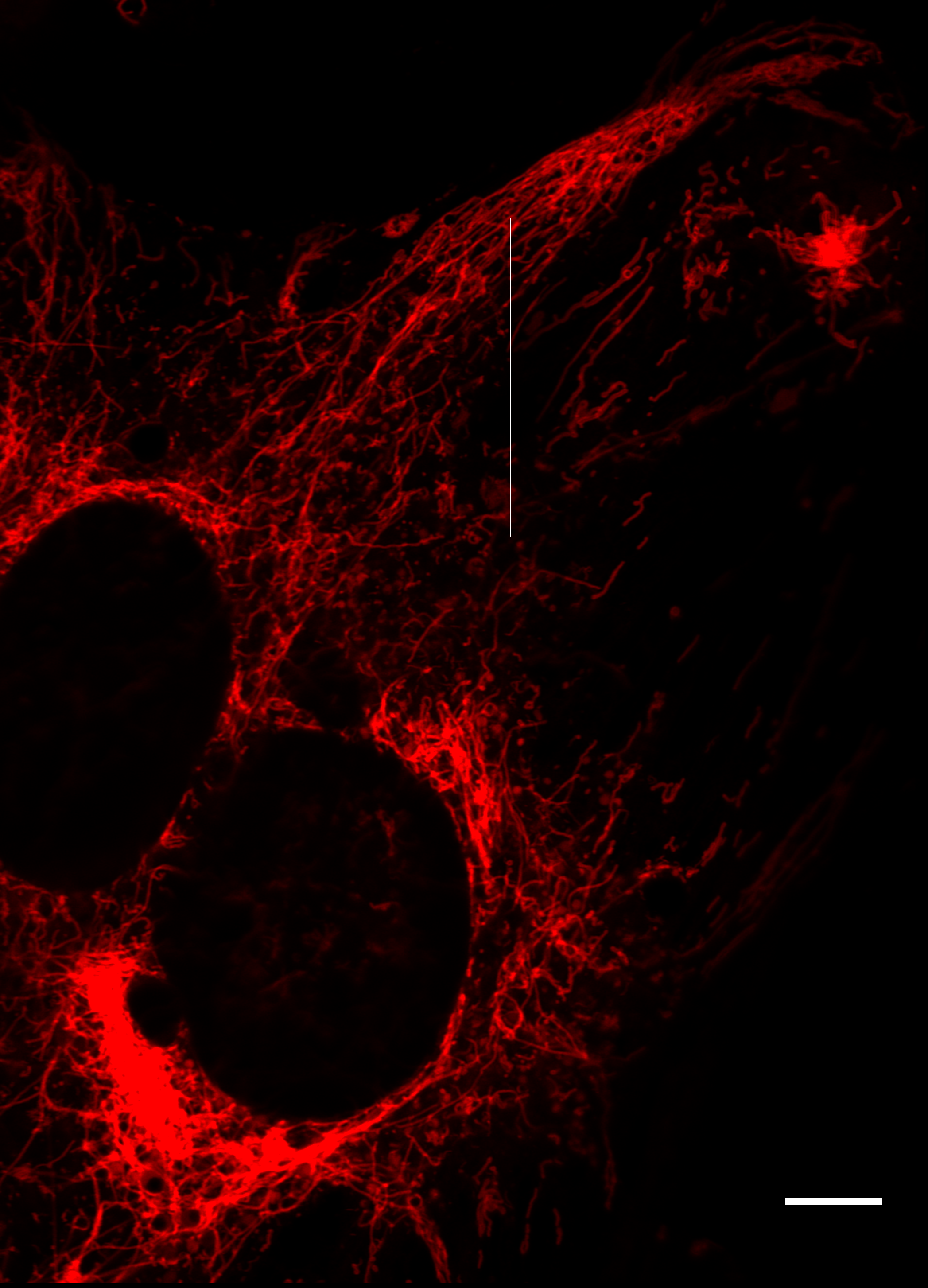

Supplement: Supplementary file 4 — Supplementary Data 1 [file 41467_2020_16572_MOESM4_ESM.zip › Super-Res. Images/Fig. 5d/NCLX KO+NE/NCLX KO t-1.tif]

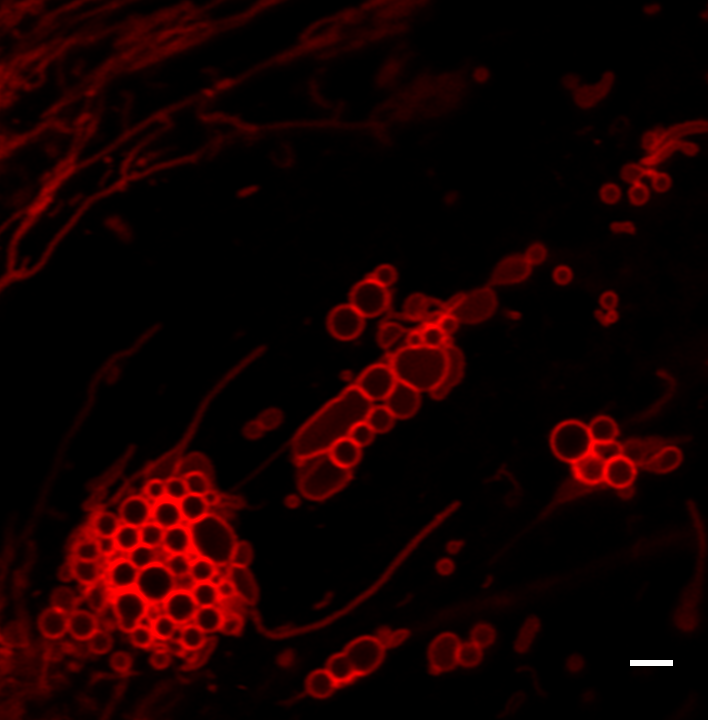

Supplement: Supplementary file 4 — Supplementary Data 1 [file 41467_2020_16572_MOESM4_ESM.zip › Super-Res. Images/Fig. 5d/NCLX KO+NE/NCLX KO t-2 ZOOM.tif]

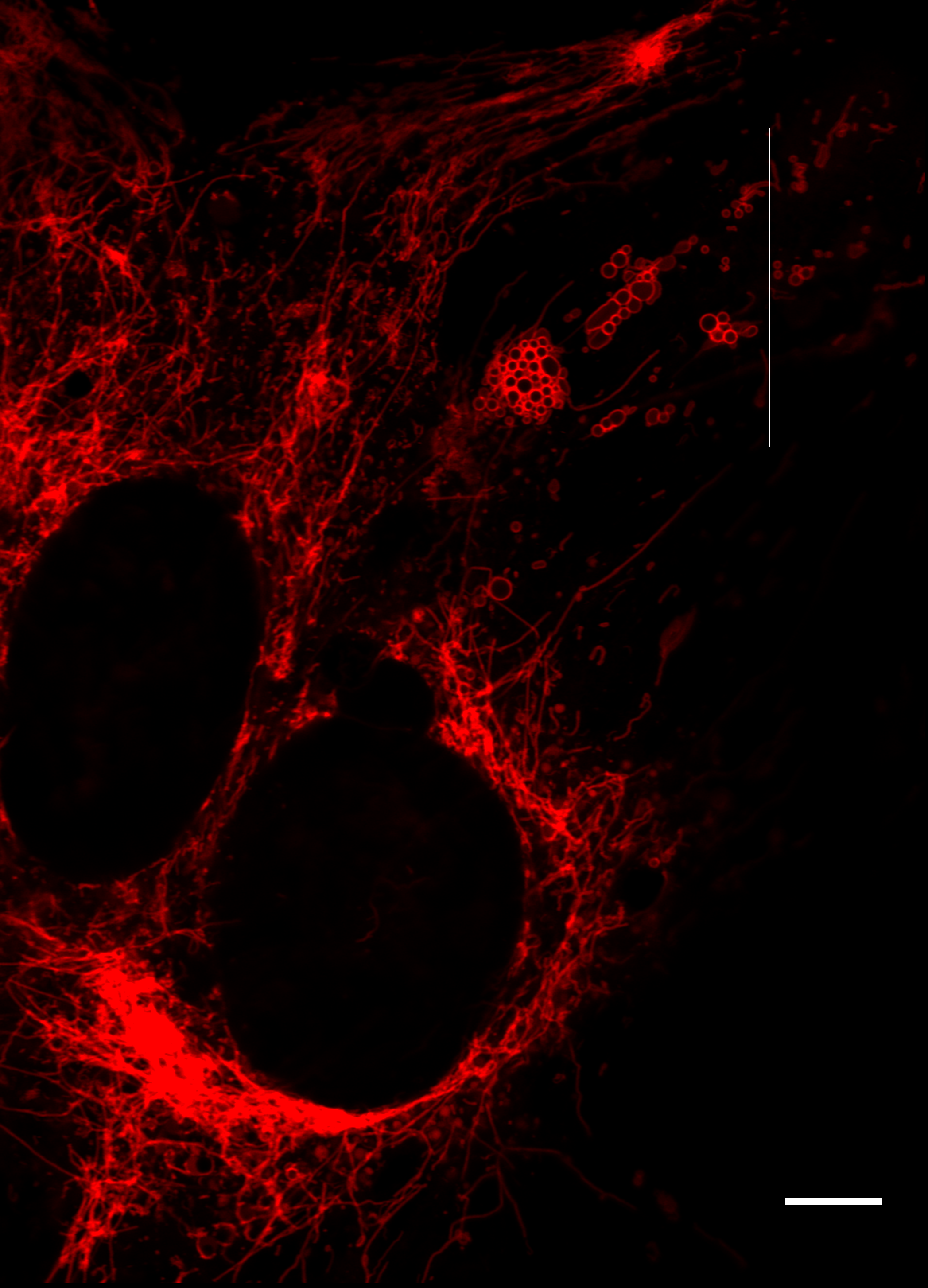

Supplement: Supplementary file 4 — Supplementary Data 1 [file 41467_2020_16572_MOESM4_ESM.zip › Super-Res. Images/Fig. 5d/NCLX KO+NE/NCLX KO t-2.tif]

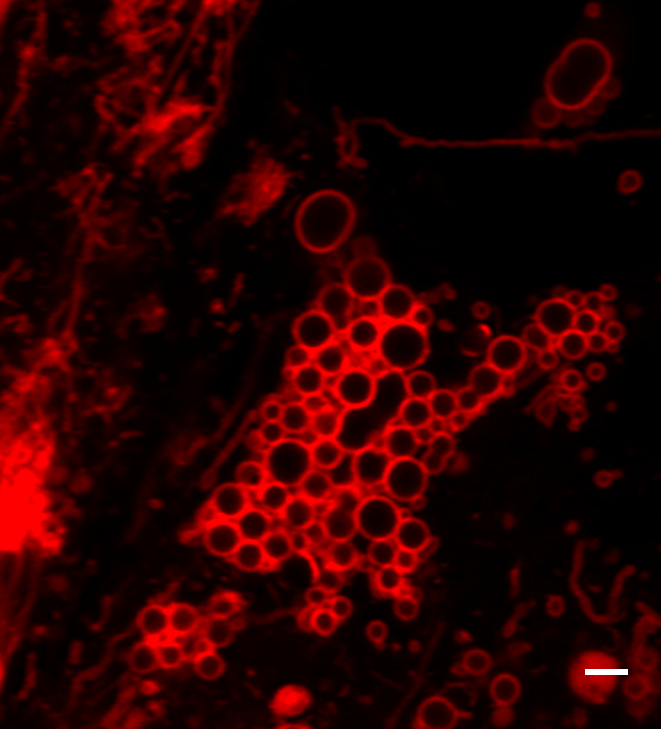

Supplement: Supplementary file 4 — Supplementary Data 1 [file 41467_2020_16572_MOESM4_ESM.zip › Super-Res. Images/Fig. 5d/NCLX KO+NE/NCLX KO t-3 ZOOM.tif]

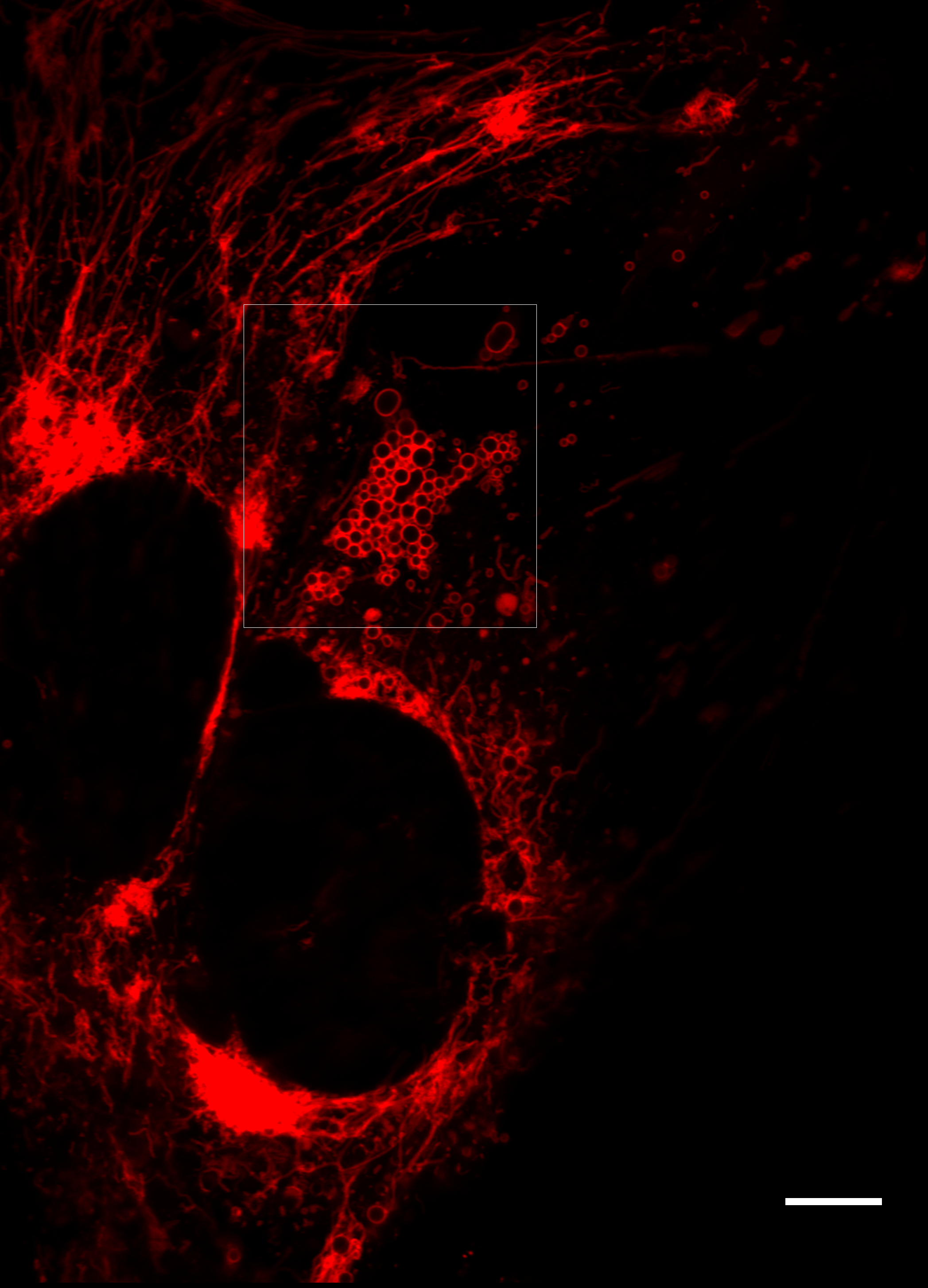

Supplement: Supplementary file 4 — Supplementary Data 1 [file 41467_2020_16572_MOESM4_ESM.zip › Super-Res. Images/Fig. 5d/NCLX KO+NE/NCLX KO t-3.tif]

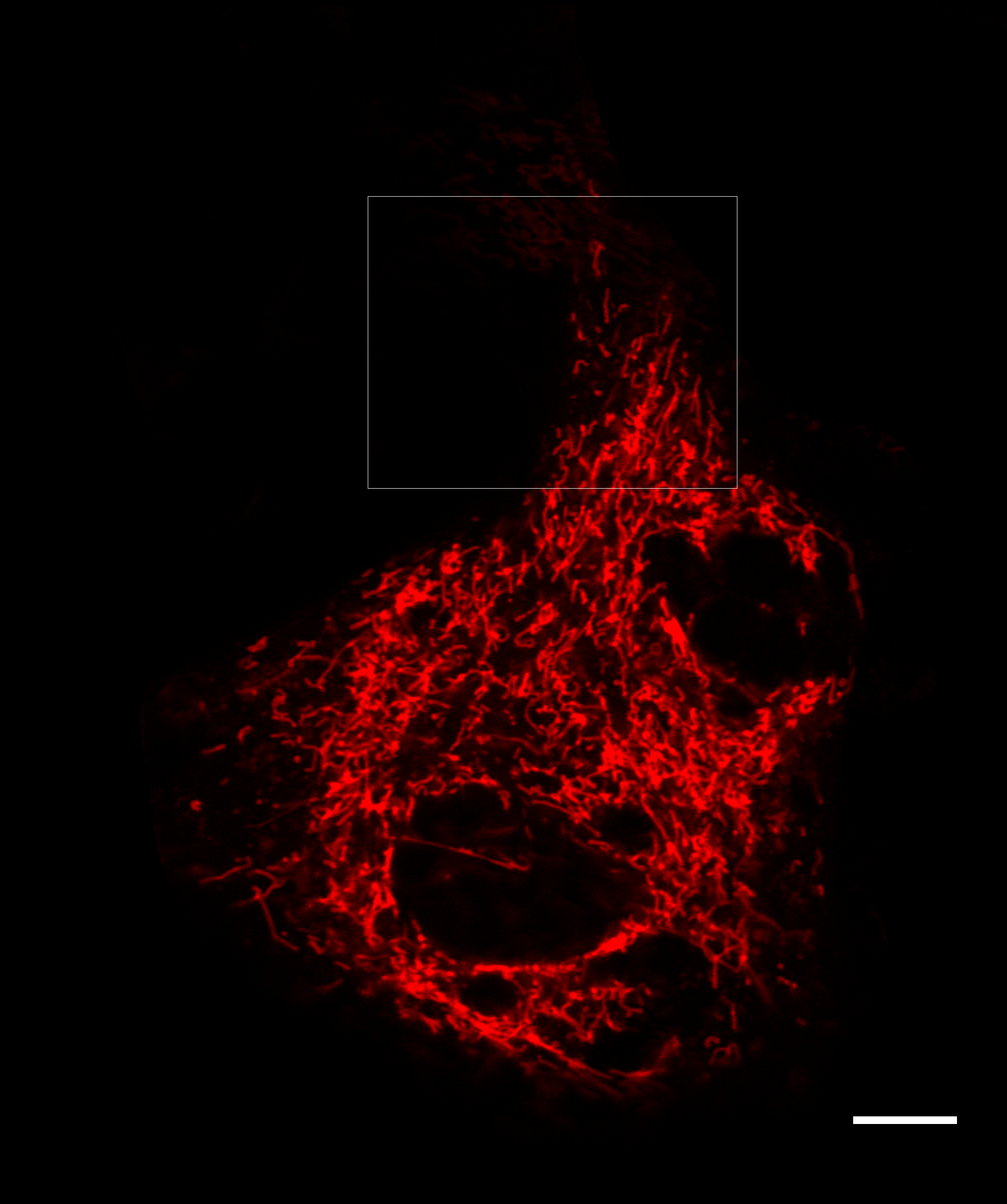

Supplement: Supplementary file 4 — Supplementary Data 1 [file 41467_2020_16572_MOESM4_ESM.zip › Super-Res. Images/Fig. 5d/WT+NE/WT-t1.tif]

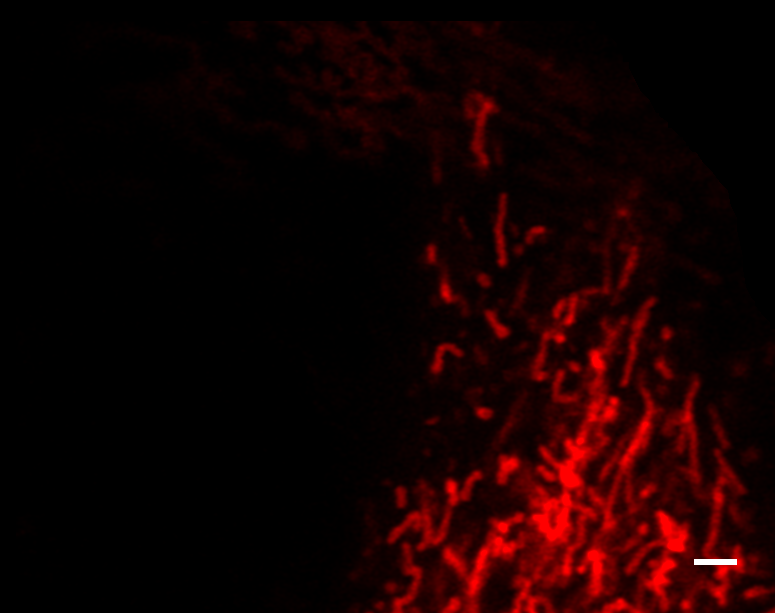

Supplement: Supplementary file 4 — Supplementary Data 1 [file 41467_2020_16572_MOESM4_ESM.zip › Super-Res. Images/Fig. 5d/WT+NE/WT-t1-ROI-ZOOM.tif]

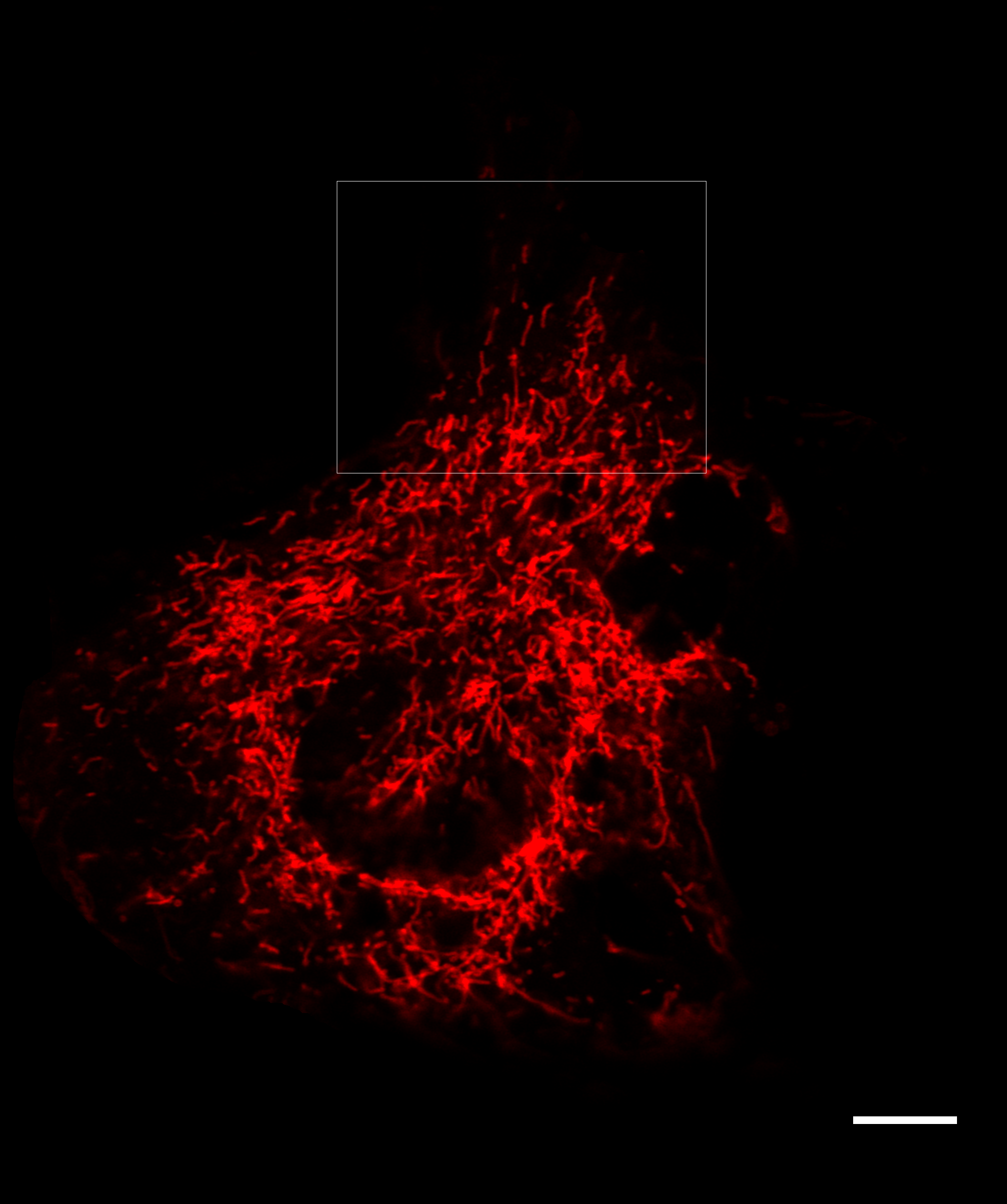

Supplement: Supplementary file 4 — Supplementary Data 1 [file 41467_2020_16572_MOESM4_ESM.zip › Super-Res. Images/Fig. 5d/WT+NE/WT-t2.tif]

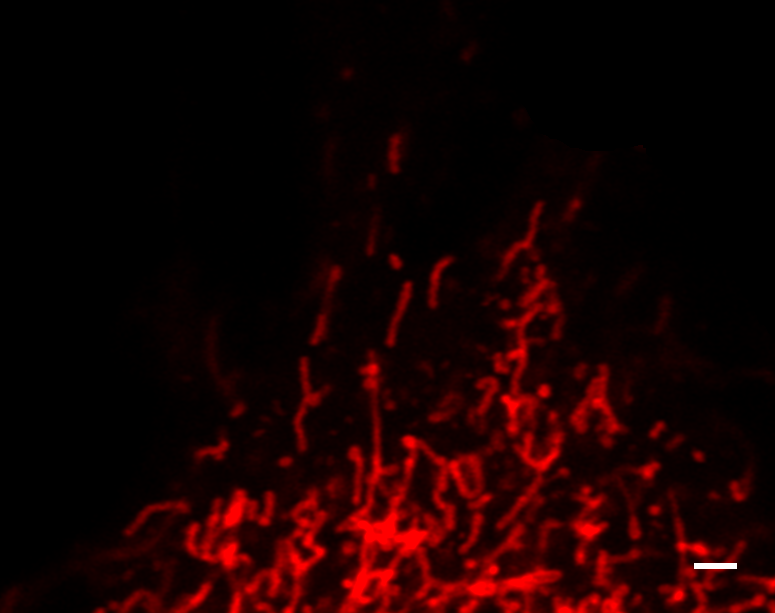

Supplement: Supplementary file 4 — Supplementary Data 1 [file 41467_2020_16572_MOESM4_ESM.zip › Super-Res. Images/Fig. 5d/WT+NE/WT-t2-ROI-ZOOM.tif]

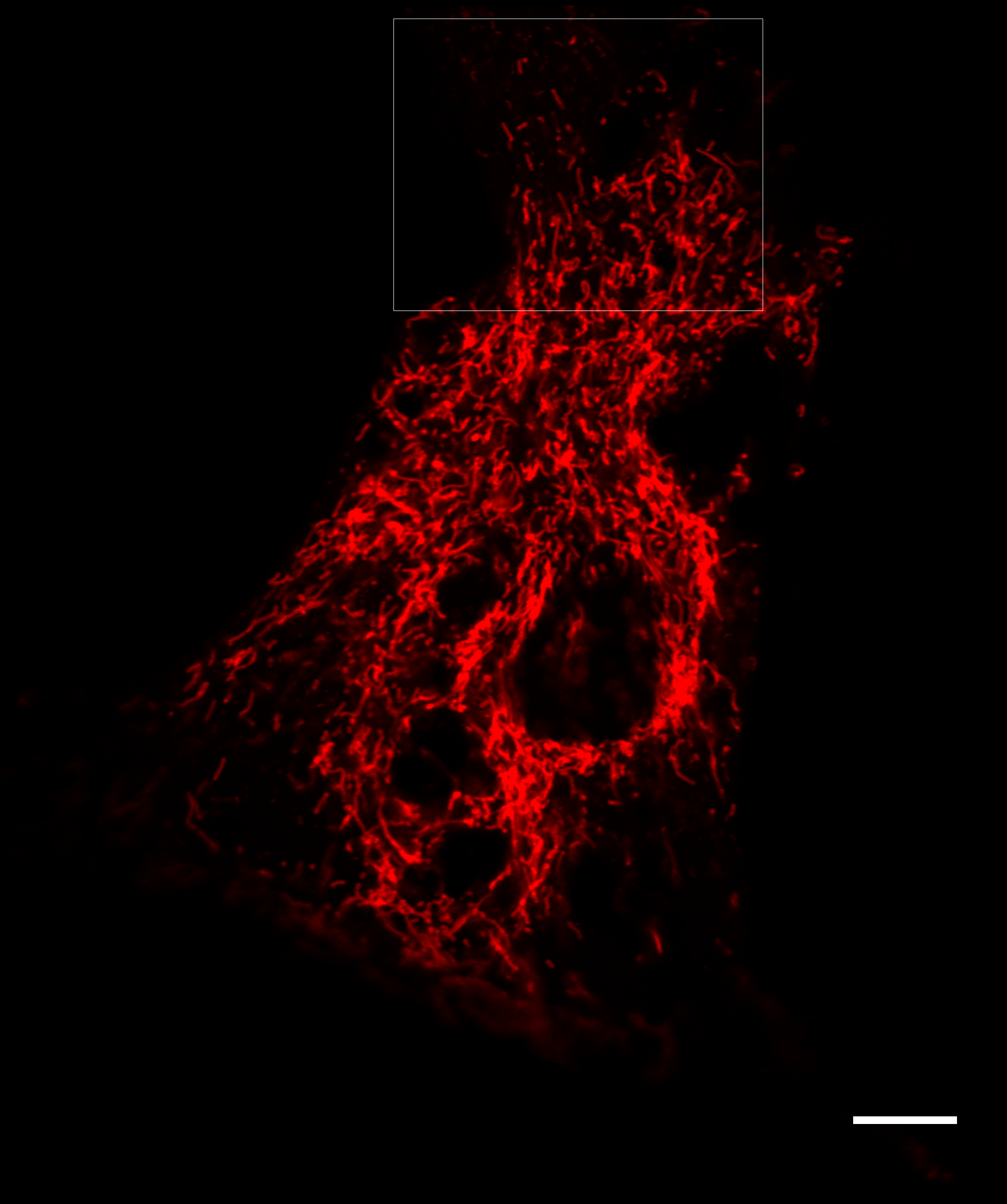

Supplement: Supplementary file 4 — Supplementary Data 1 [file 41467_2020_16572_MOESM4_ESM.zip › Super-Res. Images/Fig. 5d/WT+NE/WT-t3.tif]

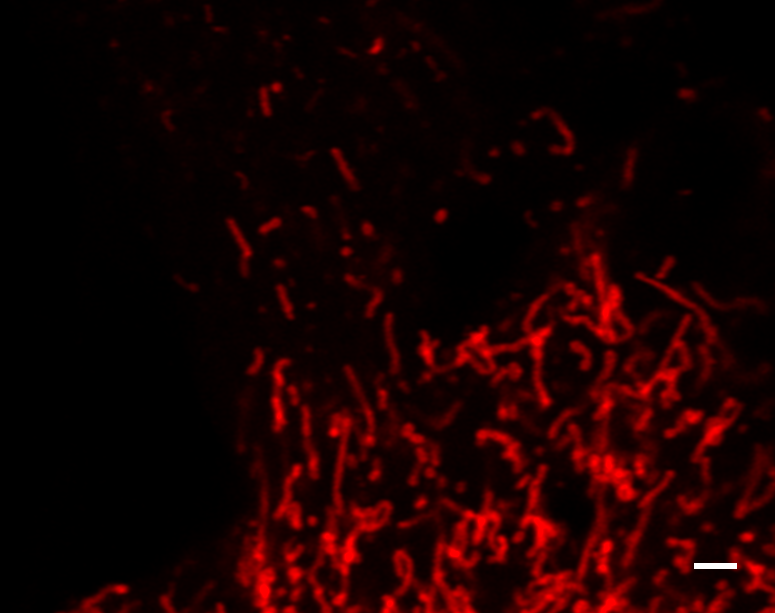

Supplement: Supplementary file 4 — Supplementary Data 1 [file 41467_2020_16572_MOESM4_ESM.zip › Super-Res. Images/Fig. 5d/WT+NE/WT-t3-ROI-ZOOM.tif]

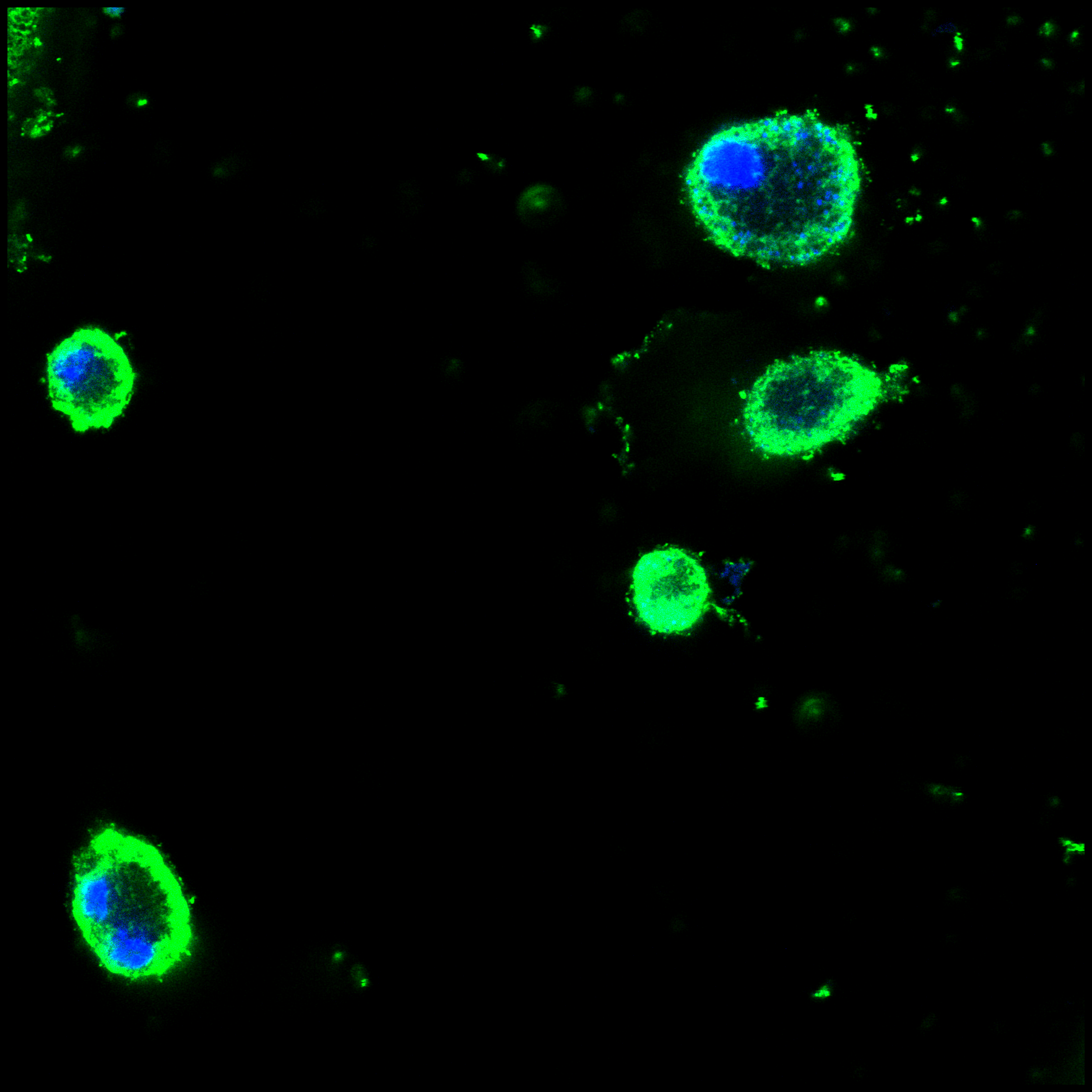

Supplement: Supplementary file 4 — Supplementary Data 1 [file 41467_2020_16572_MOESM4_ESM.zip › Super-Res. Images/Fig. 7h/Stimulated NCLX KO (post 72h)/Composite.tif]

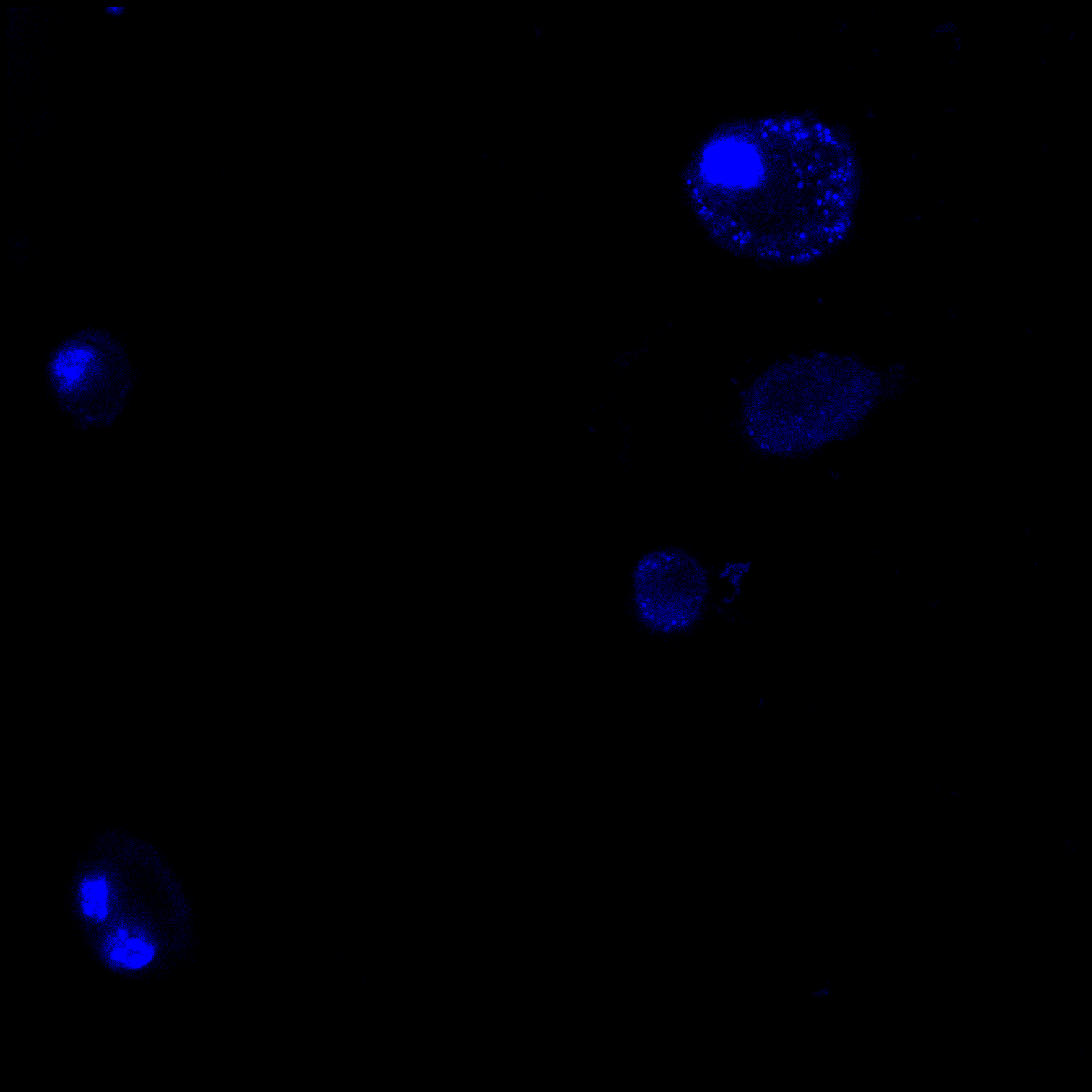

Supplement: Supplementary file 4 — Supplementary Data 1 [file 41467_2020_16572_MOESM4_ESM.zip › Super-Res. Images/Fig. 7h/Stimulated NCLX KO (post 72h)/DAPI.tif]

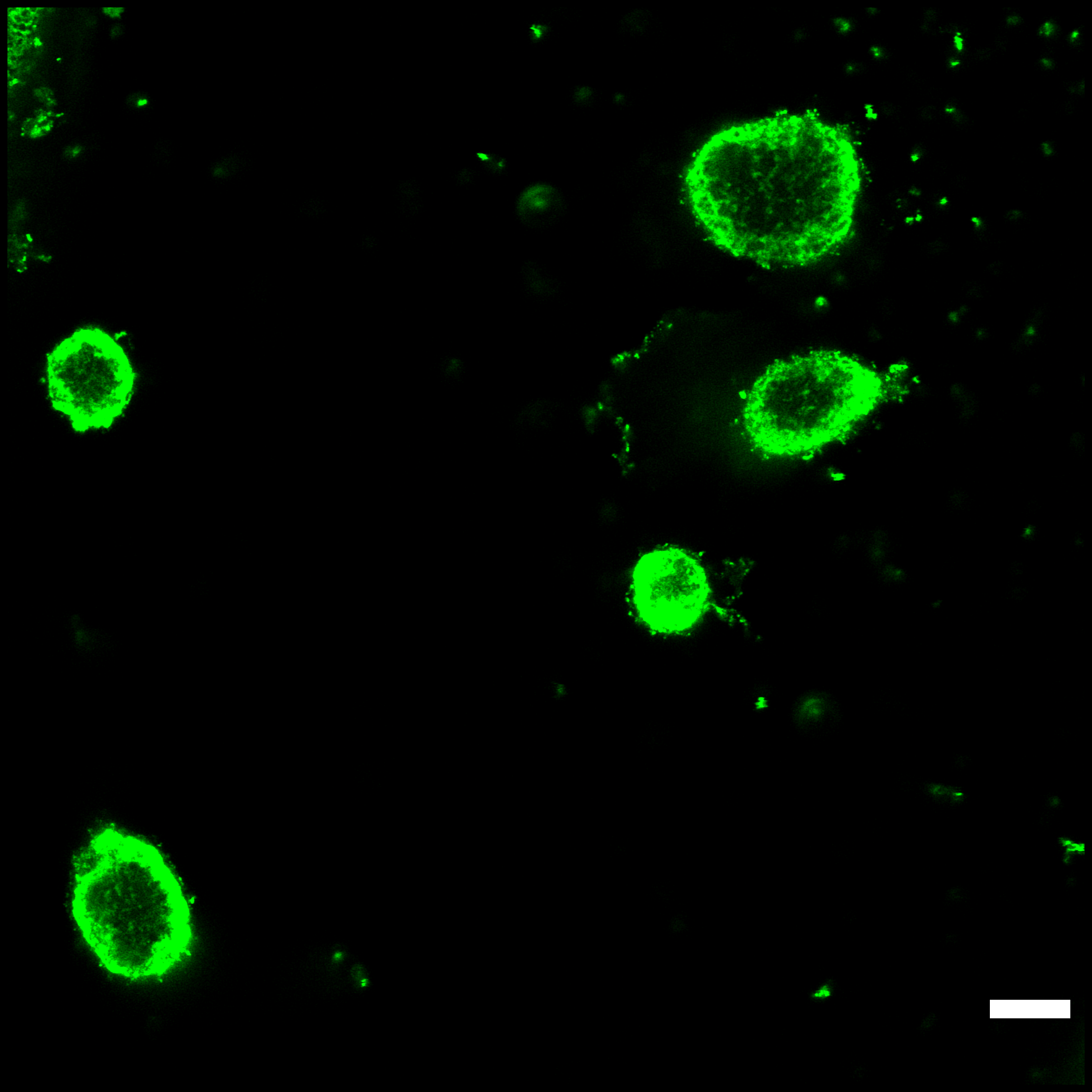

Supplement: Supplementary file 4 — Supplementary Data 1 [file 41467_2020_16572_MOESM4_ESM.zip › Super-Res. Images/Fig. 7h/Stimulated NCLX KO (post 72h)/TOM20.tif]

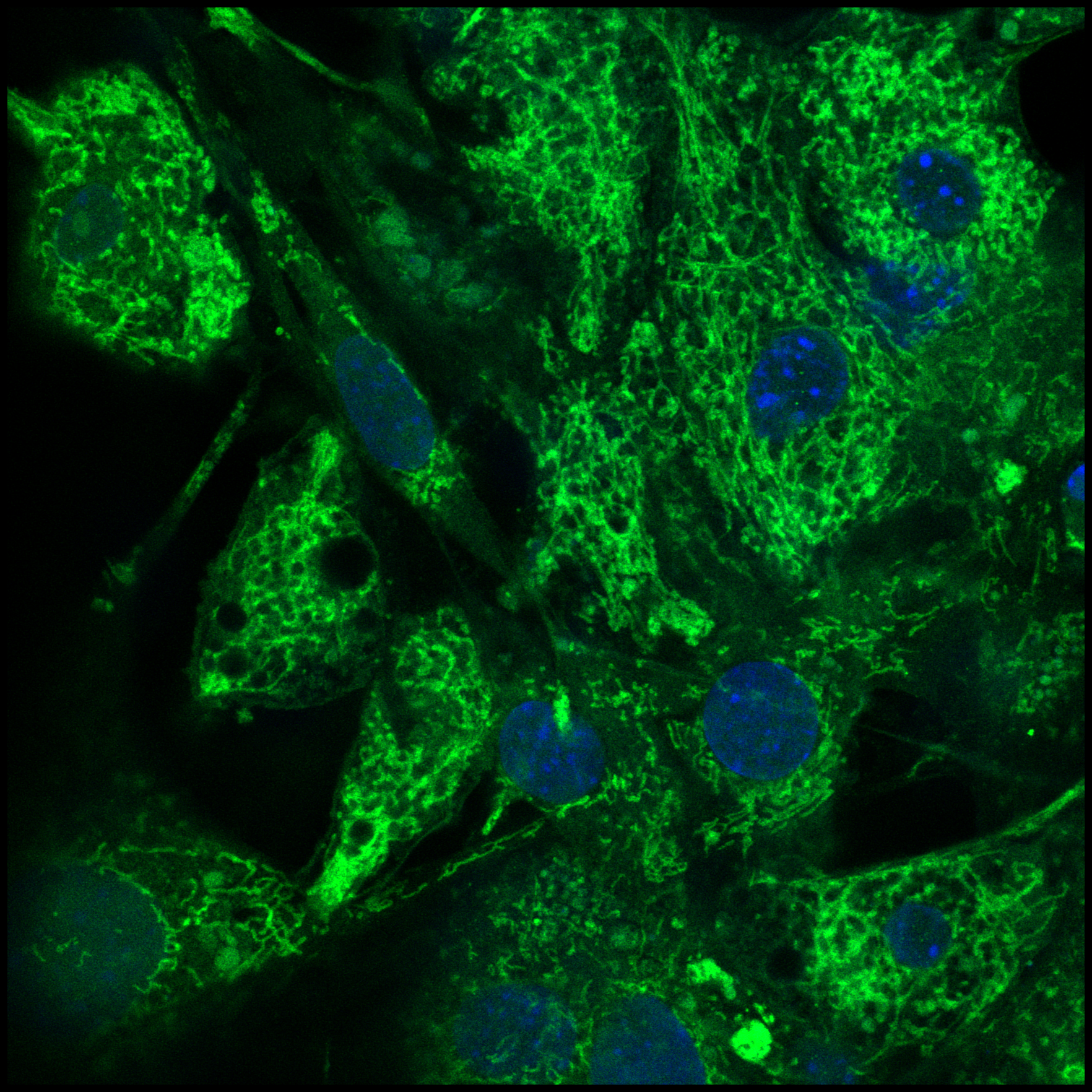

Supplement: Supplementary file 4 — Supplementary Data 1 [file 41467_2020_16572_MOESM4_ESM.zip › Super-Res. Images/Fig. 7h/Stimulated NCLX KO+NIM811 (post 72h)/Composite.tif]

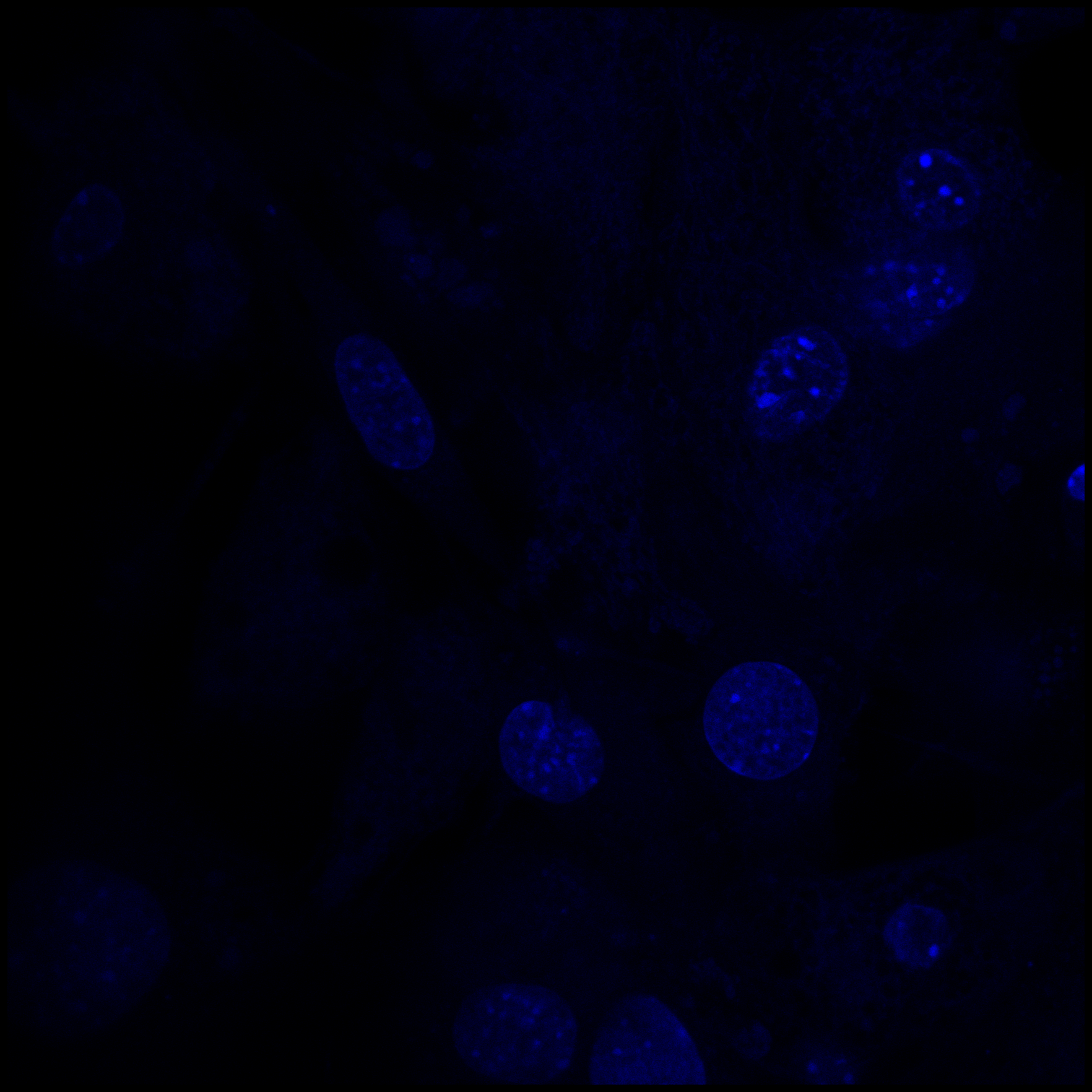

Supplement: Supplementary file 4 — Supplementary Data 1 [file 41467_2020_16572_MOESM4_ESM.zip › Super-Res. Images/Fig. 7h/Stimulated NCLX KO+NIM811 (post 72h)/DAPI.tif]

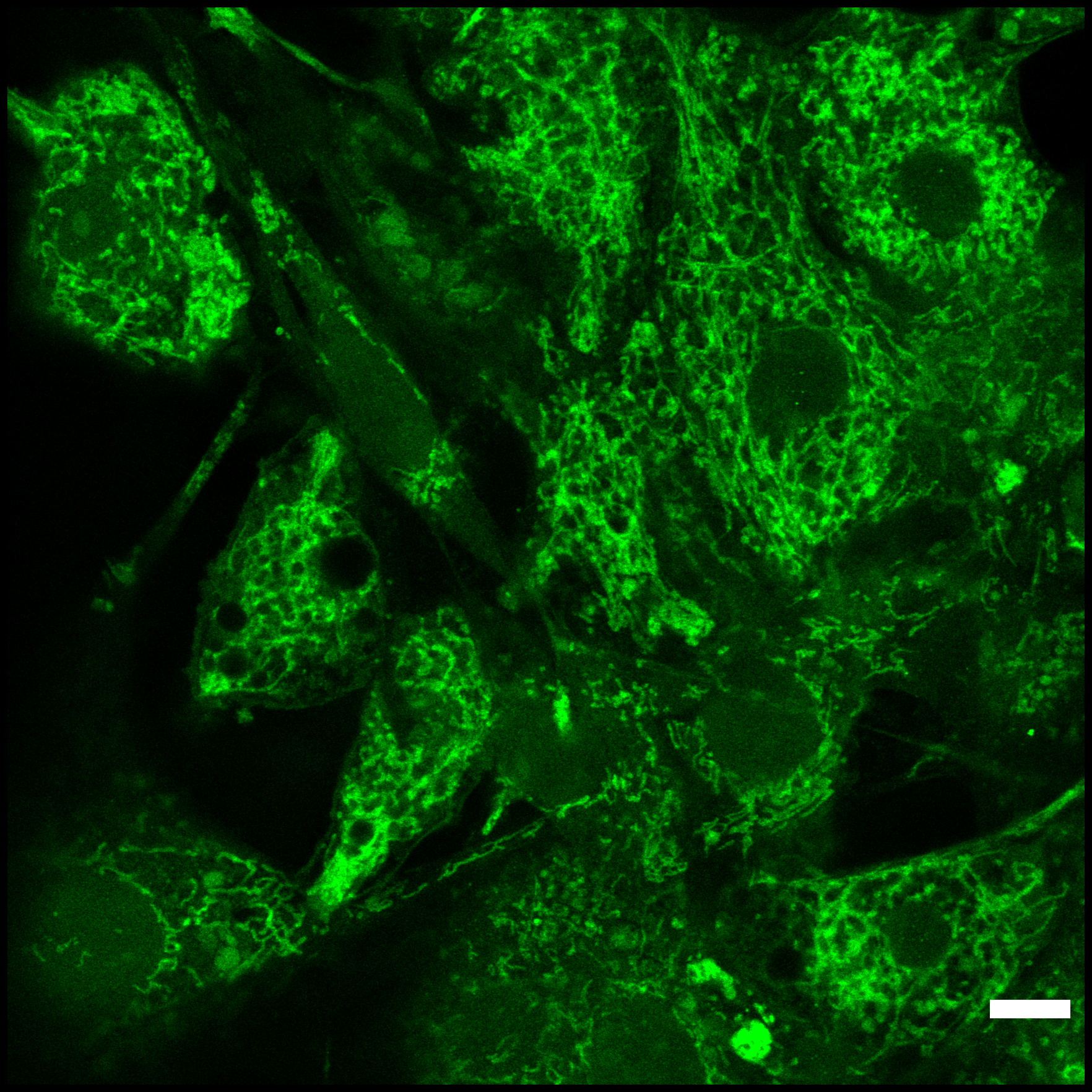

Supplement: Supplementary file 4 — Supplementary Data 1 [file 41467_2020_16572_MOESM4_ESM.zip › Super-Res. Images/Fig. 7h/Stimulated NCLX KO+NIM811 (post 72h)/TOM20.tif]

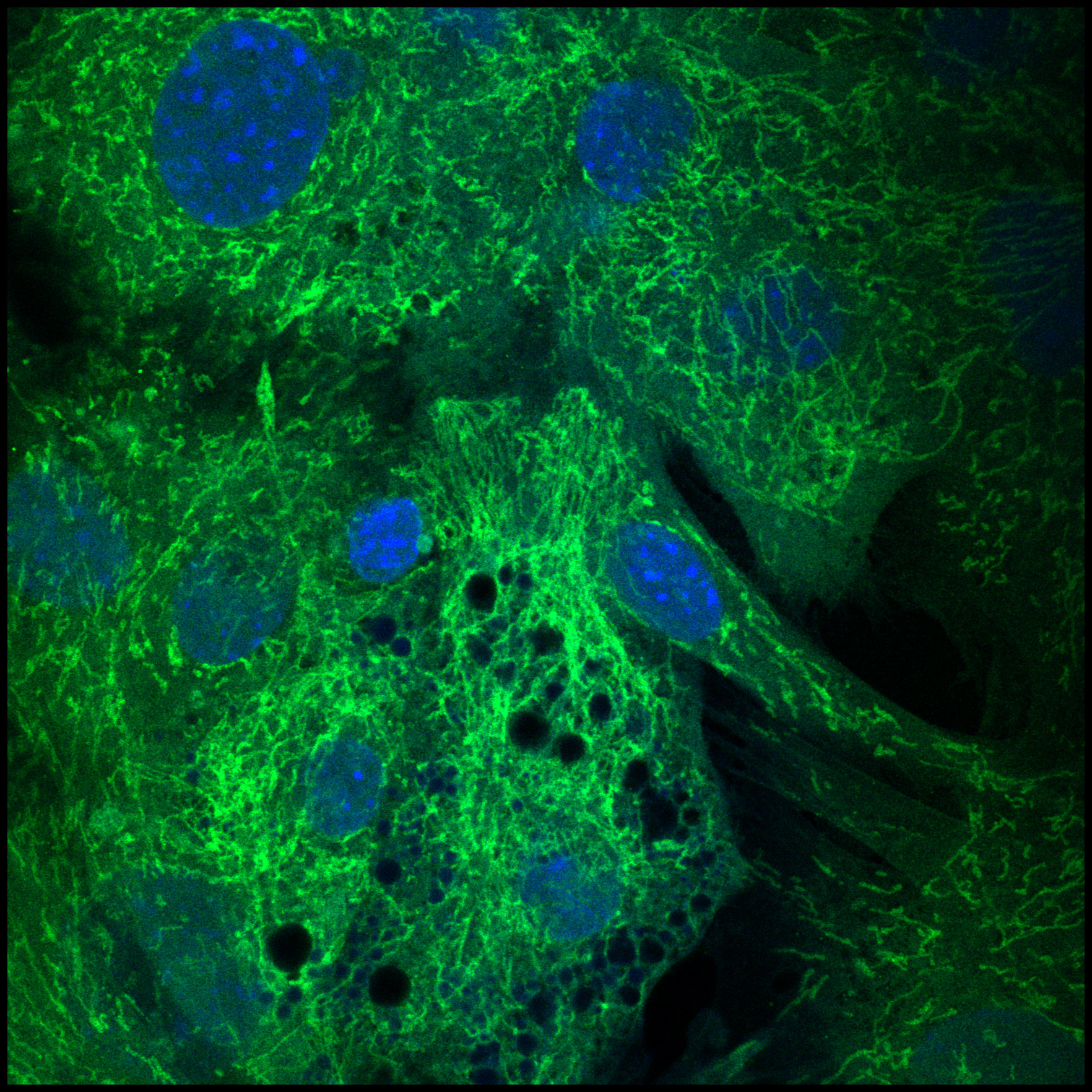

Supplement: Supplementary file 4 — Supplementary Data 1 [file 41467_2020_16572_MOESM4_ESM.zip › Super-Res. Images/Fig. 7h/Stimulated WT (post 72h)/Composite.tif]

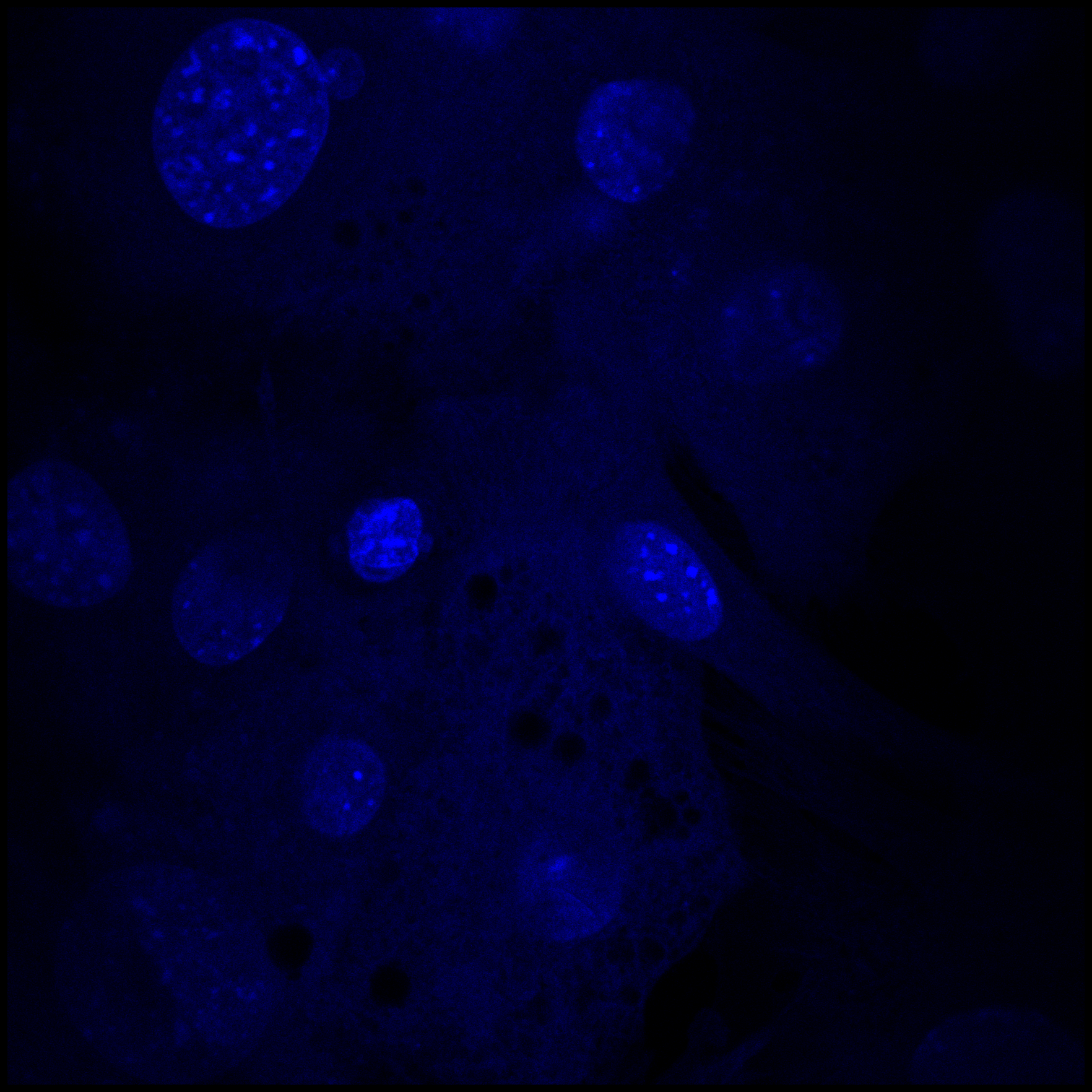

Supplement: Supplementary file 4 — Supplementary Data 1 [file 41467_2020_16572_MOESM4_ESM.zip › Super-Res. Images/Fig. 7h/Stimulated WT (post 72h)/DAPI.tif]

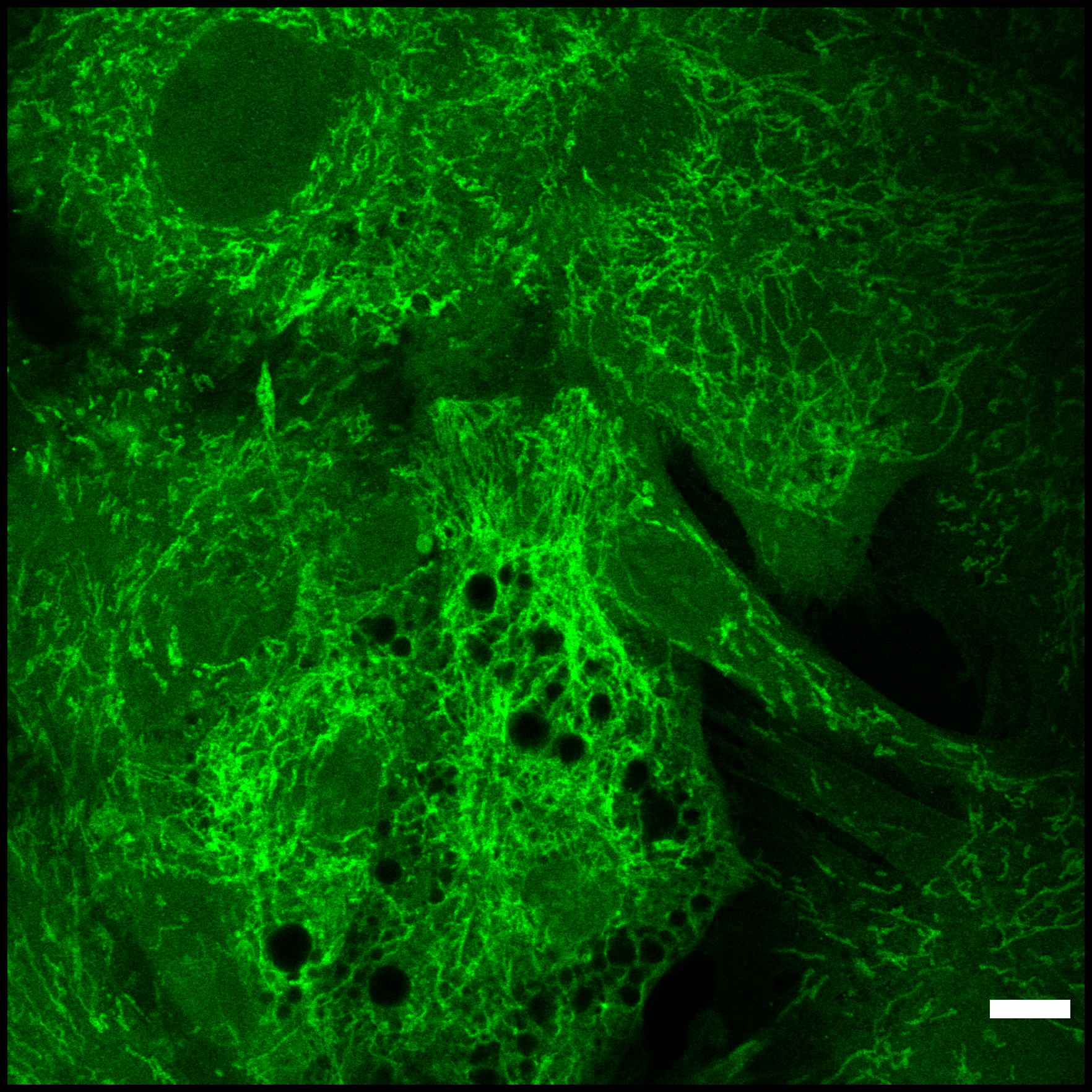

Supplement: Supplementary file 4 — Supplementary Data 1 [file 41467_2020_16572_MOESM4_ESM.zip › Super-Res. Images/Fig. 7h/Stimulated WT (post 72h)/TOM20.tif]

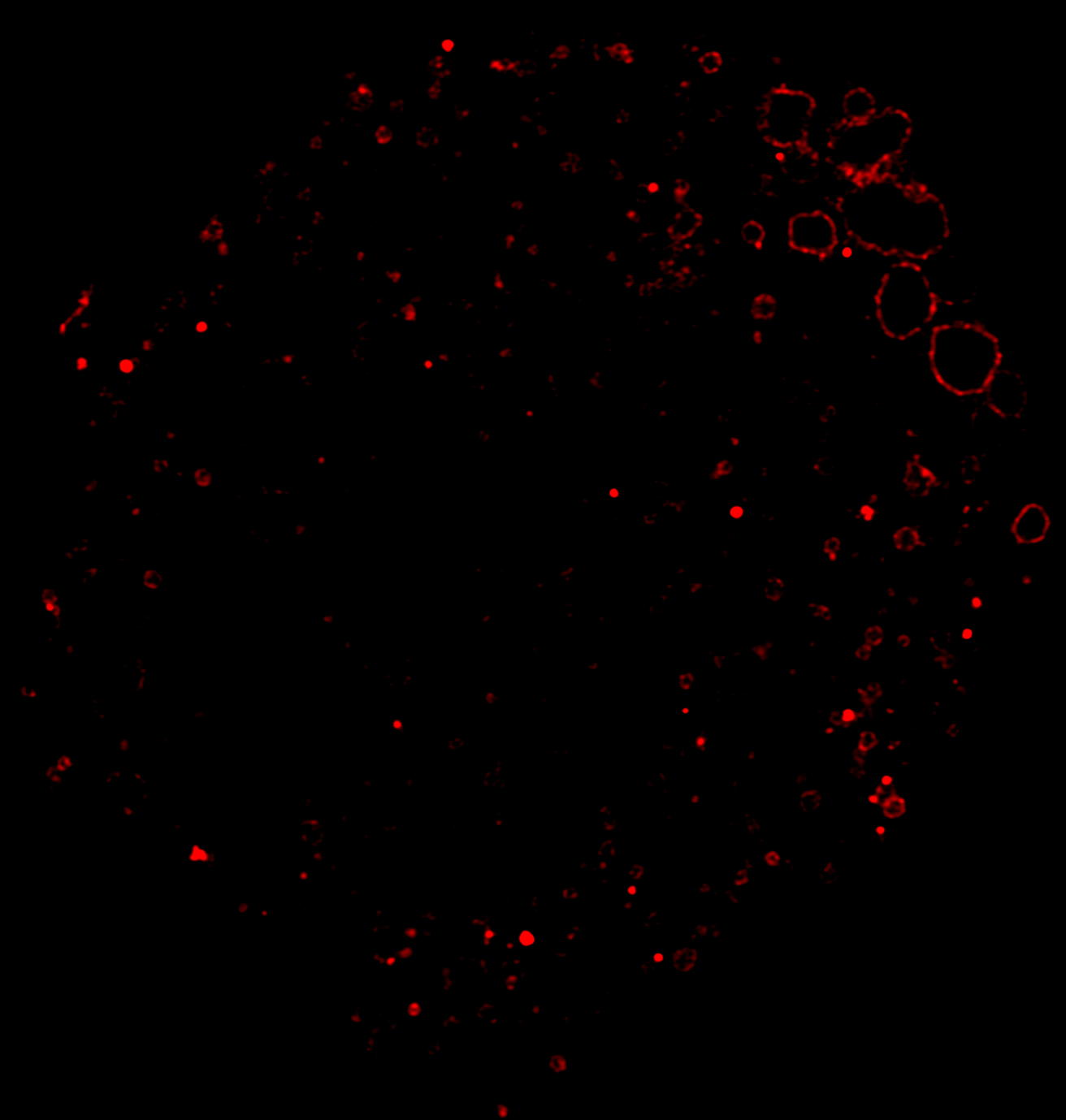

Supplement: Supplementary file 4 — Supplementary Data 1 [file 41467_2020_16572_MOESM4_ESM.zip › Super-Res. Images/Sup. Fig. 7a/NCLX KO+NE (Cyt. c).tif]

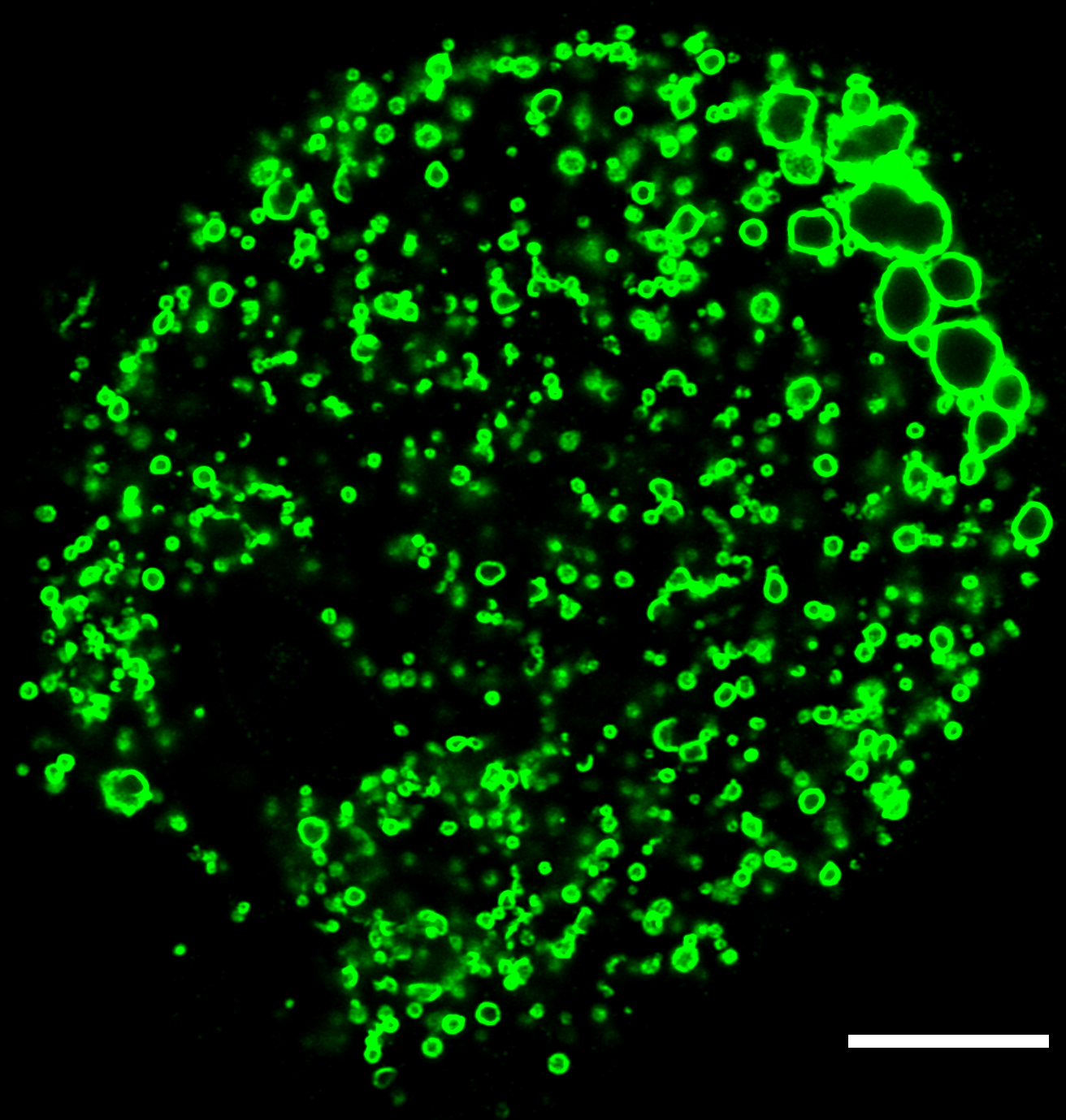

Supplement: Supplementary file 4 — Supplementary Data 1 [file 41467_2020_16572_MOESM4_ESM.zip › Super-Res. Images/Sup. Fig. 7a/NCLX KO+NE (TOM20).tif]

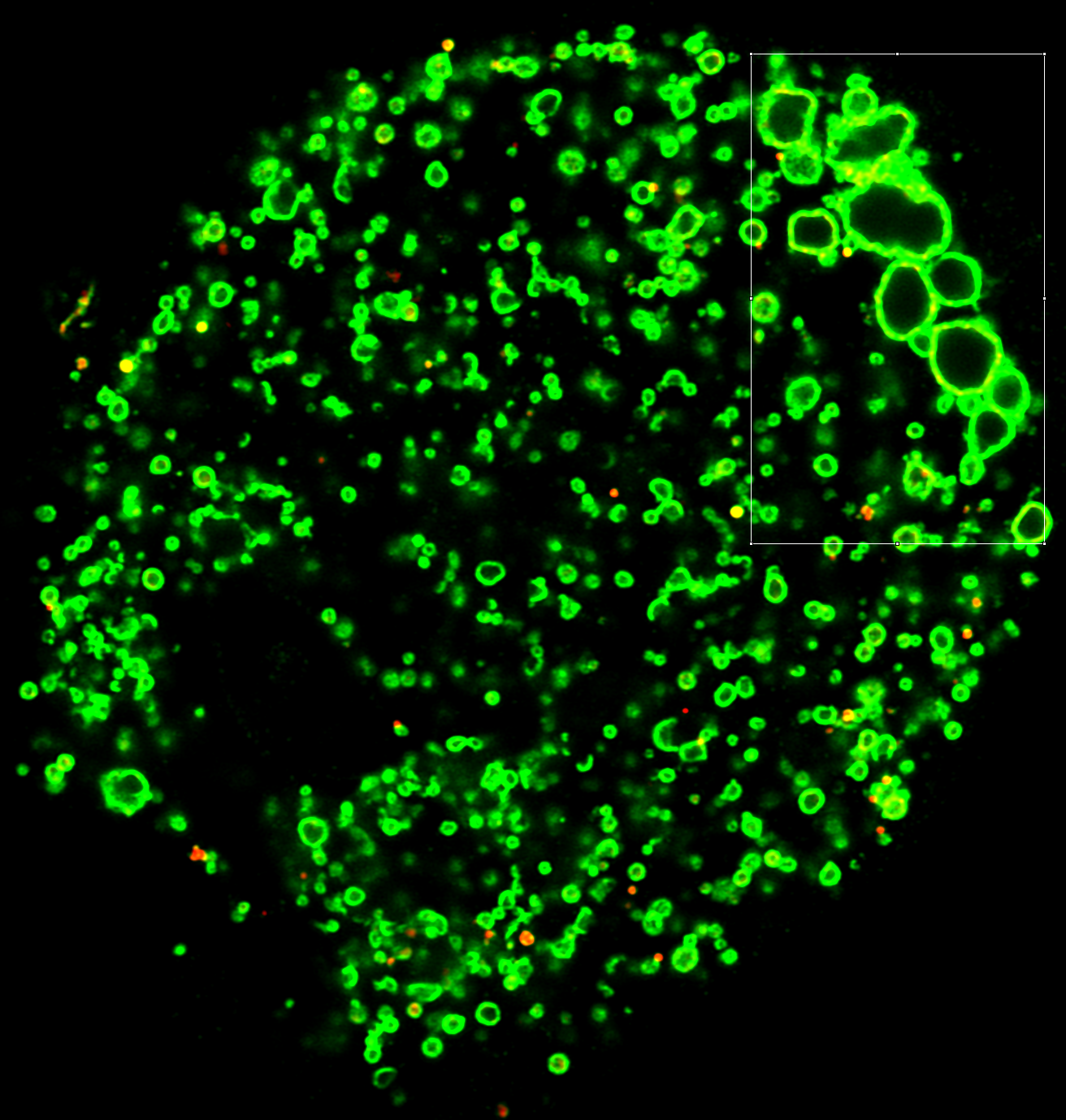

Supplement: Supplementary file 4 — Supplementary Data 1 [file 41467_2020_16572_MOESM4_ESM.zip › Super-Res. Images/Sup. Fig. 7a/NCLX KO+NE Composite.tif]

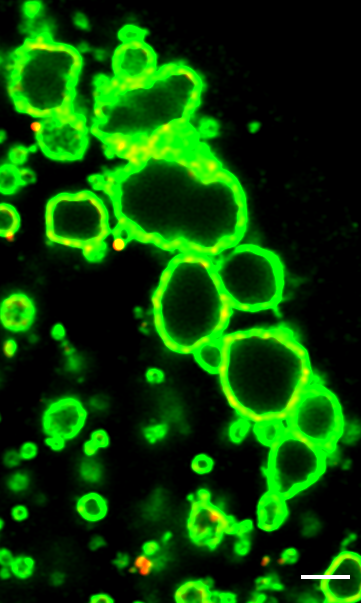

Supplement: Supplementary file 4 — Supplementary Data 1 [file 41467_2020_16572_MOESM4_ESM.zip › Super-Res. Images/Sup. Fig. 7a/NCLX KO+NE Zoom image.tif]

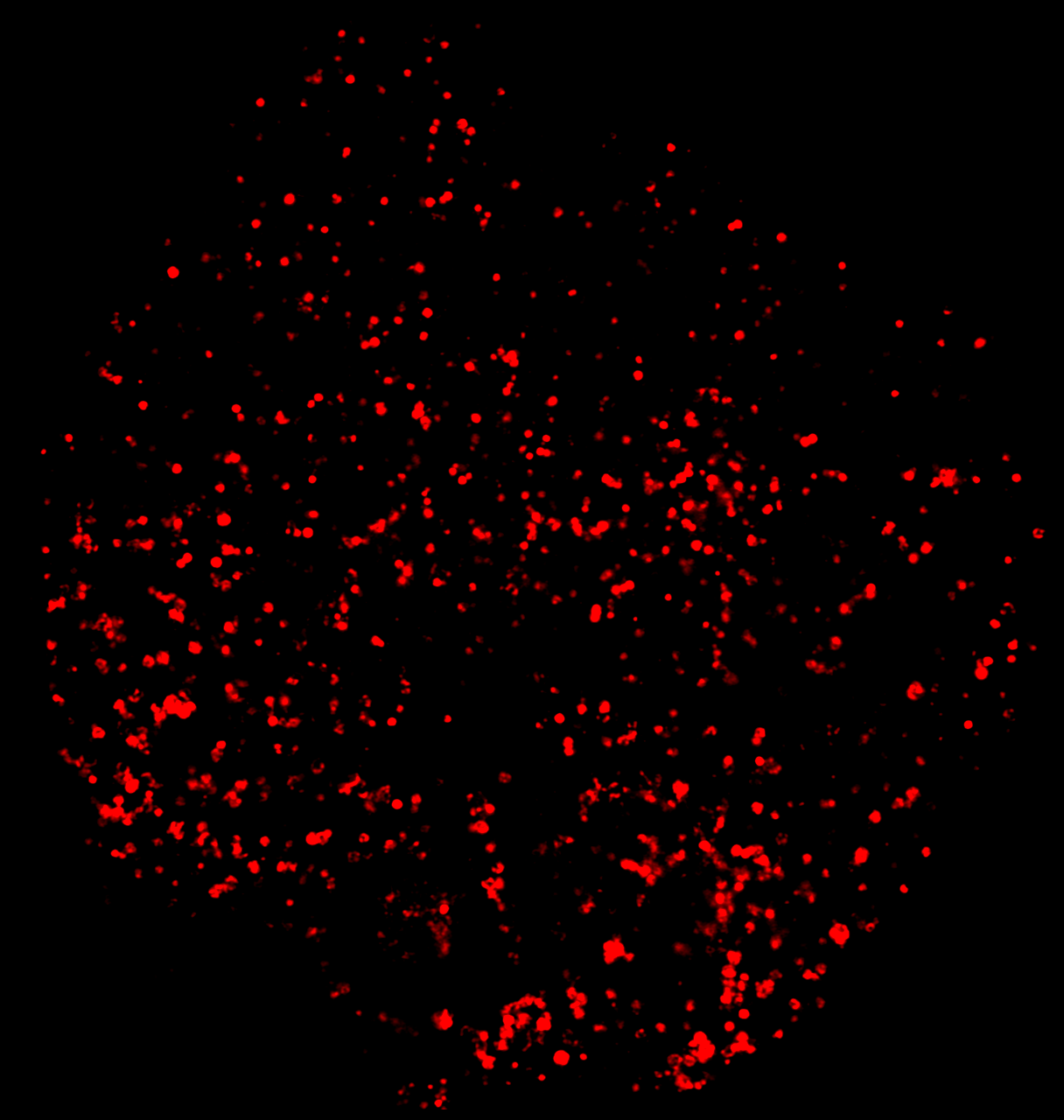

Supplement: Supplementary file 4 — Supplementary Data 1 [file 41467_2020_16572_MOESM4_ESM.zip › Super-Res. Images/Sup. Fig. 7b/NCLX KO+NE (Cyt. c).tif]

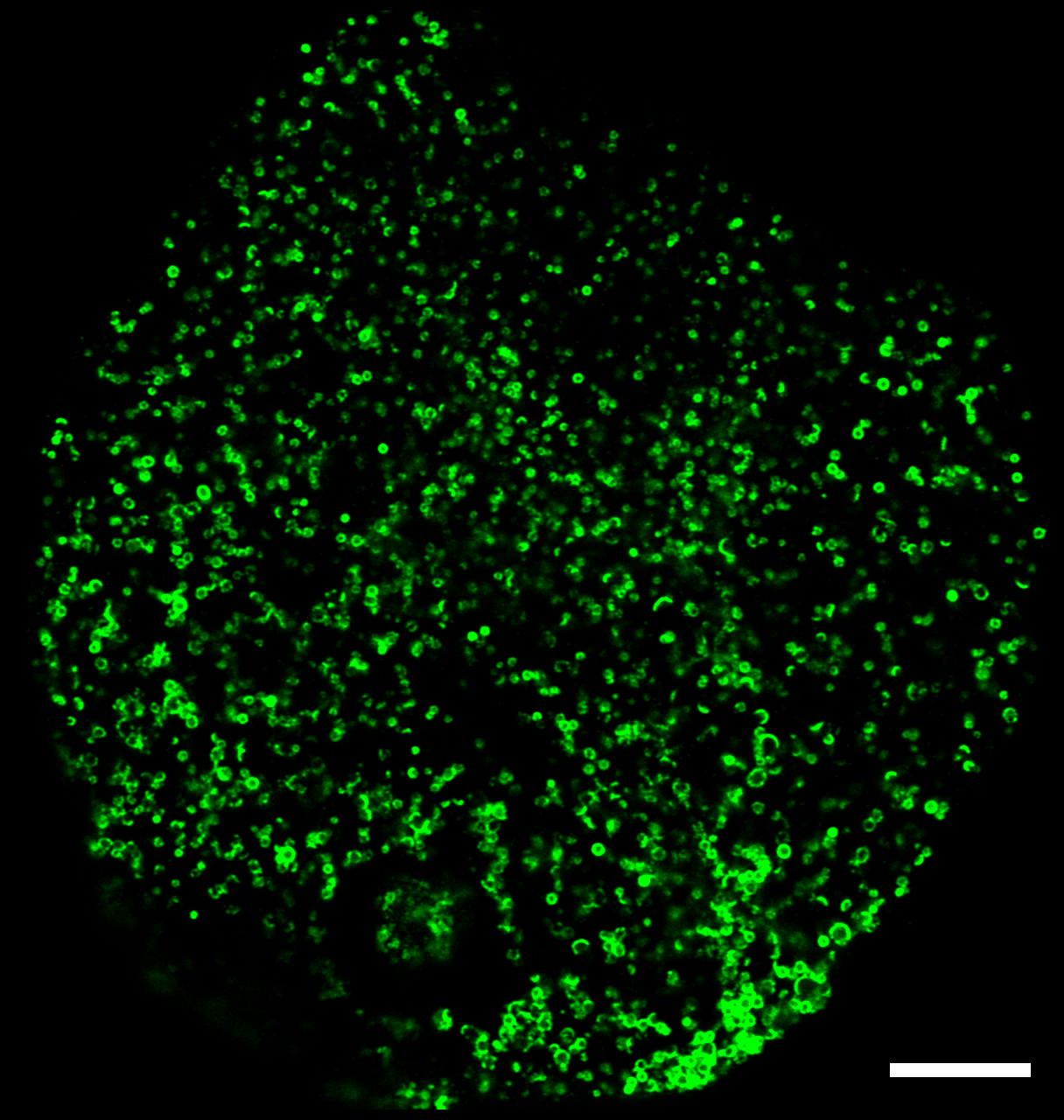

Supplement: Supplementary file 4 — Supplementary Data 1 [file 41467_2020_16572_MOESM4_ESM.zip › Super-Res. Images/Sup. Fig. 7b/NCLX KO+NE (TOM20).tif]

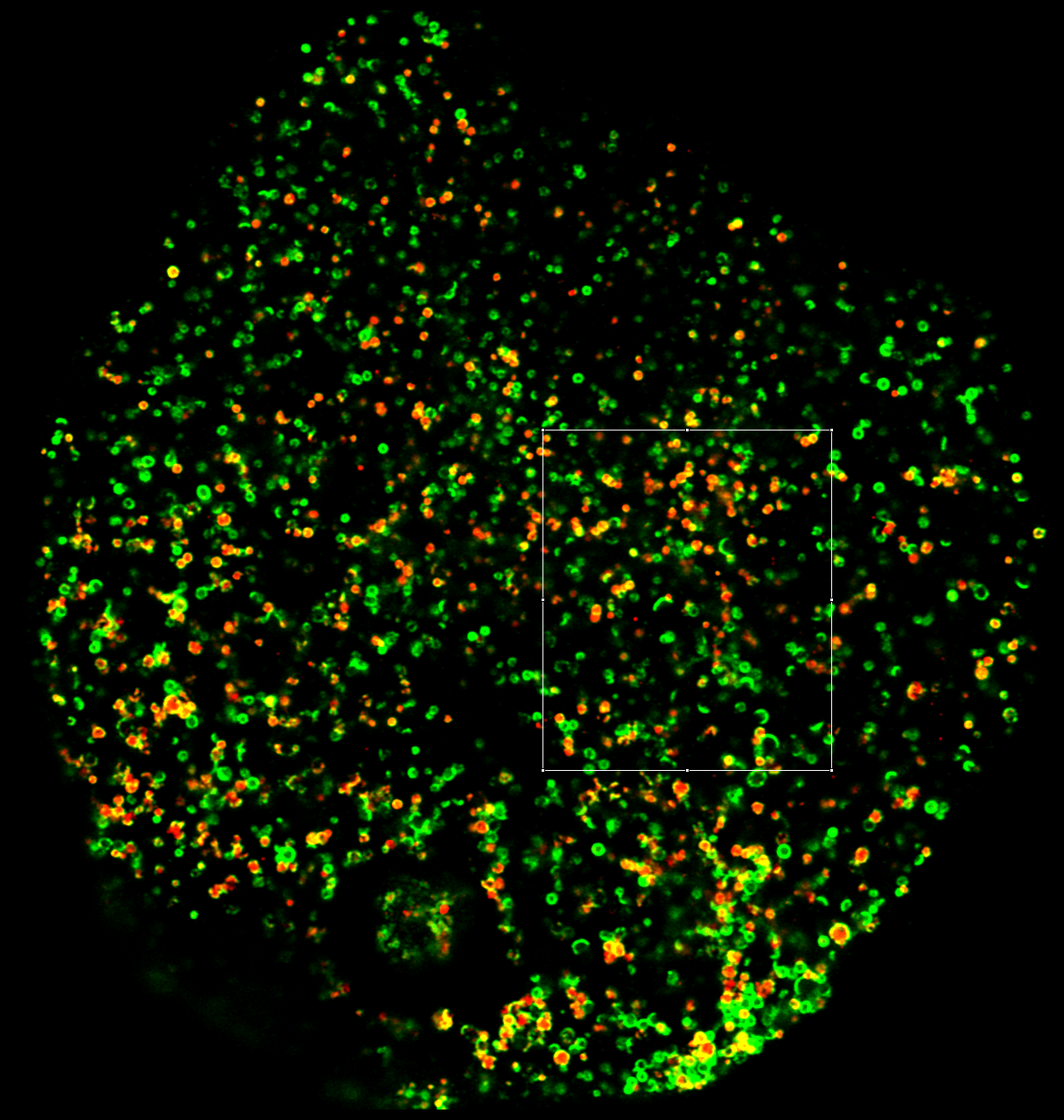

Supplement: Supplementary file 4 — Supplementary Data 1 [file 41467_2020_16572_MOESM4_ESM.zip › Super-Res. Images/Sup. Fig. 7b/NCLX KO+NE- Composite.tif]

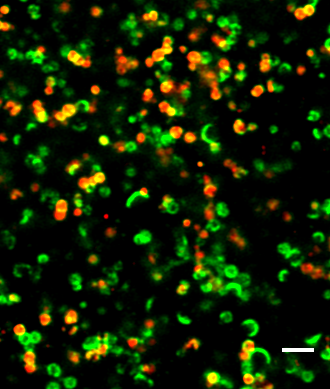

Supplement: Supplementary file 4 — Supplementary Data 1 [file 41467_2020_16572_MOESM4_ESM.zip › Super-Res. Images/Sup. Fig. 7b/NCLX KO+NE Zoom.tif]

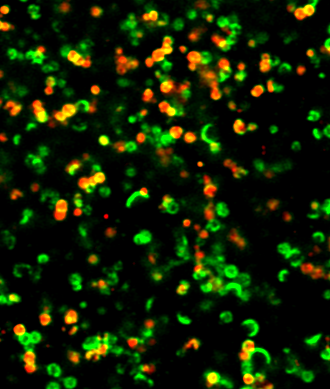

Supplement: Supplementary file 4 — Supplementary Data 1 [file 41467_2020_16572_MOESM4_ESM.zip › Super-Res. Images/Sup. Fig. 7b/ZOOM-84.tif]
