# Supplementary material for: NCLX prevents cell death during adrenergic activation of the brown adipose tissue
Source: Nat Commun. 2020 Jul 3;11:3347. doi: 10.1038/s41467-020-16572-3 (PMC7334226; doi:10.1038/s41467-020-16572-3)
Supplement: Supplementary file 8 — Reporting Summary [file 41467_2020_16572_MOESM8_ESM.pdf]

nature research

Corresponding author(s): Orian S. Shirihai

Reporting Summary

Nature Research wishes to improve the reproducibility of the work that we publish. This form provides structure for consistency and transparency in reporting. For further information on Nature Research policies, see Authors & Referees and the Editorial Policy Checklist.

Statistical parameters

When statistical analyses are reported, confirm that the following items are present in the relevant location (e.g. figure legend, table legend, main text, or Methods section).

n/a	Confirmed	
☐	☒	The exact sample size (*n*) for each experimental group/condition, given as a discrete number and unit of measurement
☐	☒	An indication of whether measurements were taken from distinct samples or whether the same sample was measured repeatedly
☐	☒	The statistical test(s) used AND whether they are one- or two-sided<br>*Only common tests should be described solely by name; describe more complex techniques in the Methods section.*
☒	☐	A description of all covariates tested
☐	☒	A description of any assumptions or corrections, such as tests of normality and adjustment for multiple comparisons
☐	☒	A full description of the statistics including central tendency (e.g. means) or other basic estimates (e.g. regression coefficient) AND variation (e.g. standard deviation) or associated estimates of uncertainty (e.g. confidence intervals)
☐	☒	For null hypothesis testing, the test statistic (e.g. *F*, *t*, *r*) with confidence intervals, effect sizes, degrees of freedom and *P* value noted<br>*Give P values as exact values whenever suitable.*
☒	☐	For Bayesian analysis, information on the choice of priors and Markov chain Monte Carlo settings
☒	☐	For hierarchical and complex designs, identification of the appropriate level for tests and full reporting of outcomes
☒	☐	Estimates of effect sizes (e.g. Cohen's *d*, Pearson's *r*), indicating how they were calculated
☐	☒	Clearly defined error bars<br>*State explicitly what error bars represent (e.g. SD, SE, CI)*

Our web collection on statistics for biologists may be useful.

Software and code

Policy information about availability of computer code

Data collection	We have used the following softwares to collect imaging data:<br>-Black Zen Software v2.3 for LSM880 microscope image acquisition.<br>-Imaging WorkBench v6.0 software for Ca+2 imaging.<br>-Imagelab v6.0 for Western Blot acquisition and analysis.
Data analysis	We have used the following softwares to analyze data in this study:<br>-Excel 2016.<br>- FIJI ImageJ, https://fiji.sc (NIH)<br>- Graphpad Prism v6.0 (GraphPad Software, Inc).<br>-Kaleidagraph v4.5.<br>-Amide v1.0.4.<br>-QuPath v0.1.2.

For manuscripts utilizing custom algorithms or software that are central to the research but not yet described in published literature, software must be made available to editors/reviewers upon request. We strongly encourage code deposition in a community repository (e.g. GitHub). See the Nature Research guidelines for submitting code & software for further information.

Data

Policy information about availability of data

All manuscripts must include a data availability statement. This statement should provide the following information, where applicable:

- Accession codes, unique identifiers, or web links for publicly available datasets
- A list of figures that have associated raw data
- A description of any restrictions on data availability

All datasets generated in the current manuscript are available on reasonable request.

Field-specific reporting

Please select the best fit for your research. If you are not sure, read the appropriate sections before making your selection.

☒ Life sciences ☐ Behavioural & social sciences ☐ Ecological, evolutionary & environmental sciences

For a reference copy of the document with all sections, see nature.com/authors/policies/ReportingSummary-flat.pdf

Life sciences study design

All studies must disclose on these points even when the disclosure is negative.

Sample size	The sample size was chosen according to our previous experience with this technique and other references performing similar studies (n=3-10 for in vivo studies). Per IACUC compliance, we used the minimal sample size that would give statistical significant differences for the parameters measured.<br>Sample sizes for each animal study has been included in the figure legends.
Data exclusions	Exclusion criteria include presence, after veterinary assessment, of infections, ulcerative dermatitis, bladder enlargements or other common diseases in this mouse strain, not specific to the genetic manipulation. For image analysis data exclusion criteria is described in the methods and applied equally to all groups tested.
Replication	The experimental findings were reproduced and all replication attempts were successful.
Randomization	While we needed the genotypes to compare littermates and therefore experimental groups were created by identifying the mice. however, some in vivo measurements were done in the absence of the genotype information at the time of data collection and randomization of known genotypes was applied by personnel who are unfamiliar with the study. Moreover, Core and other investigators did not have the genotype information when performing staining and imaging.
Blinding	Blinding was performed in in vivo and in vitro tests, including Metabolic chambers, histology, PET-CT and imaging.<br>personnel participating and cores helping performing these assays did not have the genotypes or any assumptions. Other assays were not blinded.

Reporting for specific materials, systems and methods

Materials & experimental systems

n/a	Involved in the study
☒	☐ Unique biological materials
☐	☒ Antibodies
☒	☐ Eukaryotic cell lines
☒	☐ Palaeontology
☐	☒ Animals and other organisms
☒	☐ Human research participants

Methods

n/a	Involved in the study
☒	☐ ChIP-seq
☒	☐ Flow cytometry
☒	☐ MRI-based neuroimaging

Antibodies

Antibodies used	1. Total OXPHOS Rodent WB Antibody Cocktail (Abcam, ab110413)<br>2. UCP1 (Abcam, ab10983)

3. Cytochrome c (Abcam, ab110325)
4. NCLX (Santa Cruz, SC-161921 and SC-161922)
5. MCU (Santa Cruz, SC-515930)
6.Tom20 (Santa Cruz, Tom20 FL-145, SC-11415)
7. PMCA (Santa Cruz, SC-271917)
8. SERCA2 (Santa Cruz, SC-376235)
9. RYR-1/2 (34C Developmental Studies Hybridoma Bank University of Iowa).
10. anti-Complex III Core2 UQCRC2 (Proteintech, 14742-1-AP)
11. anti-Complex I NDUFA9 (Abcam, ab188373)
12. Goat anti-Rabbit Alexa Fluor or 488 (Thermo, A11008)
13. Goat anti-mouse Alexa Fluor 568 (Thermo, A11004)

Validation

Abcam statement on validation (Antibodies 1-3,11): "We use application-specific criteria to pass or fail our antibodies and typically test all of our antibodies in multiple applications. These criteria include basic standards, like a specific signal and relevant controls."
Antibodies 4,5,6 were validated by the company and others (incl. our groups).
Thermo statement (Antibodies 7-8, 12-13): "Invitrogen antibodies are currently undergoing a rigorous 2-part testing approach..."
https://www.thermofisher.com/us/en/home/life-science/antibodies/invitrogen-antibody-validation.html

Animals and other organisms

Policy information about studies involving animals; ARRIVE guidelines recommended for reporting animal research

Laboratory animals

For in-vivo experiments, WT and NCLX KO mice (C57BL/6NJ-Slc8b1em1(IMPC)J/J; Jackson laboratories) were backcrossed to C57BL/6NJ (Jackson laboratories) background.
Both males and females of WT, NCLX+/- and NCLX -/- were age and gender matched (8-12 weeks old).
For in-vitro experiments, 3-4 weeks old WT and NCLX KO from 6NJ background were cultured.
Mice were maintained under controlled conditions at 22°C with a 12:12 h light:dark cycle.
Experimental procedures conducted on mice were performed in accordance with animal welfare and in compliance with other related ethical regulations. The mice studies were conducted under an approved Institutional Animal Care and Use Committee (IACUC) protocol at the University of California, Los Angeles (UCLA) and Ben-Gurion University.

Wild animals

Provide details on animals observed in or captured in the field; report species, sex and age where possible. Describe how animals were caught and transported and what happened to captive animals after the study (if killed, explain why and describe method; if released, say where and when) OR state that the study did not involve wild animals.

Field-collected samples

For laboratory work with field-collected samples, describe all relevant parameters such as housing, maintenance, temperature, photoperiod and end-of-experiment protocol OR state that the study did not involve samples collected from the field.
